# Acetylcholine-modulated plasticity in reward-driven navigation: a computational study

**DOI:** 10.1038/s41598-018-27393-2

**Published:** 2018-06-21

**Authors:** Sara Zannone, Zuzanna Brzosko, Ole Paulsen, Claudia Clopath

**Affiliations:** 1 Imperial College London, Department of Bioengineering, South Kensington Campus, London, United Kingdom; 2 University of Cambridge, Department of Physiology, Development and Neuroscience, Physiological Laboratory, Cambridge, United Kingdom

## Abstract

Neuromodulation plays a fundamental role in the acquisition of new behaviours. In previous experimental work, we showed that acetylcholine biases hippocampal synaptic plasticity towards depression, and the subsequent application of dopamine can retroactively convert depression into potentiation. We also demonstrated that incorporating this sequentially neuromodulated Spike-Timing-Dependent Plasticity (STDP) rule in a network model of navigation yields effective learning of changing reward locations. Here, we employ computational modelling to further characterize the effects of cholinergic depression on behaviour. We find that acetylcholine, by allowing learning from negative outcomes, enhances exploration over the action space. We show that this results in a variety of effects, depending on the structure of the model, the environment and the task. Interestingly, sequentially neuromodulated STDP also yields flexible learning, surpassing the performance of other reward-modulated plasticity rules.

## Introduction

In order to survive, animals have to learn to interact with their environment in an effective way. They need to acquire new information, integrate feedback and modify their behaviour accordingly. Neuromodulation is thought to play an essential role in this process: it correlates with behavioural changes and can provide feedback and information about the environment. Dopamine, for example, acts as a reward signal and a positive reinforcement of behaviour^1–3^. Acetylcholine, on the other hand, correlates with attention^4,5^, exploration^6–9^ and spatial learning in general^6,10–14^.

While the role of neuromodulation has been widely studied in the context of decision making^15^, it is still unclear exactly what neural mechanisms mediate these changes in behaviour. Research is increasingly focusing on elucidating the effects of neuromodulation on Spike-Timing-Dependent Plasticity (STDP), a form of plasticity that depends on exact spike timings. In its classic form, STDP dictates that, when a presynaptic spike precedes a postsynaptic spike, the synapse is potentiated; it is depressed if the spike order is reversed^16,17^. Classic STDP acts on a millisecond scale, a timescale much too fast to explain behavioural effects. However, experimentally, dopamine has been found to increase the window for potentiation in STDP^18–20^. In particular, we found that dopamine can potentiate hippocampal synapses that were previously active, even when applied after a delay^21,22^, bridging the gap between synaptic and behavioural timescales. This supports the concept of an eligibility trace, which has been theorized and employed in computational modelling. An eligibility trace associates actions, and the underlying patterns of neural activity, to distal rewards^23–27^. This also offers a solution to the credit assignment problem: the problem of identifying actions that lead to rewards. Although acetylcholine has been shown to modulate synaptic plasticity in both directions^28^, we found that acetylcholine biased hippocampal STDP towards depression. Interestingly, this effect could be retroactively converted into potentation by consequent application of dopamine^22^.

Building on this experimental evidence^21,22^, we have previously investigated the possible functional effects of sequentially neuromodulated plasticity (sn-Plast)^22^. Using a bottom-up approach, we incorporated this novel rule into a spiking neural network model of reward-driven navigation^29–32^. We found that sequential cholinergic and dopaminergic modulation of plasticity allows flexible learning, particularly useful in dynamic environments with changing reward locations^22^.

Here, we set out to further investigate the functional roles of sn-Plast. Inspired by experimental observations of cholinergic effects on behaviour, we examine exploration and flexibile learning in particular. In order to confirm and expand on our previous findings, we compare our rule to other types of plasticity. We show how the effects of neuromodulated STDP on behaviour depend on various model features, including state and action spaces, maze geometry and task details. This allows us to deepen our mechanistic understanding of the model, and gain some insight into the complex relationship between synaptic and behavioural learning.

## Results

We base this work on recent experimental results that shine light on how hippocampal plasticity is affected by neuromodulation. In particular, dopamine was shown to retroactively potentiate previously active synapses, even when applied after a delay^21^. This provides evidence for the existence of an eligibility trace, a mechanism formerly proposed in the reinforcement learning literature as a solution to the credit assignment problem. Acetylcholine, on the other end, was found to induce depression in active synapses, regardless of the precise spike order^22^.

**Figure 1:**
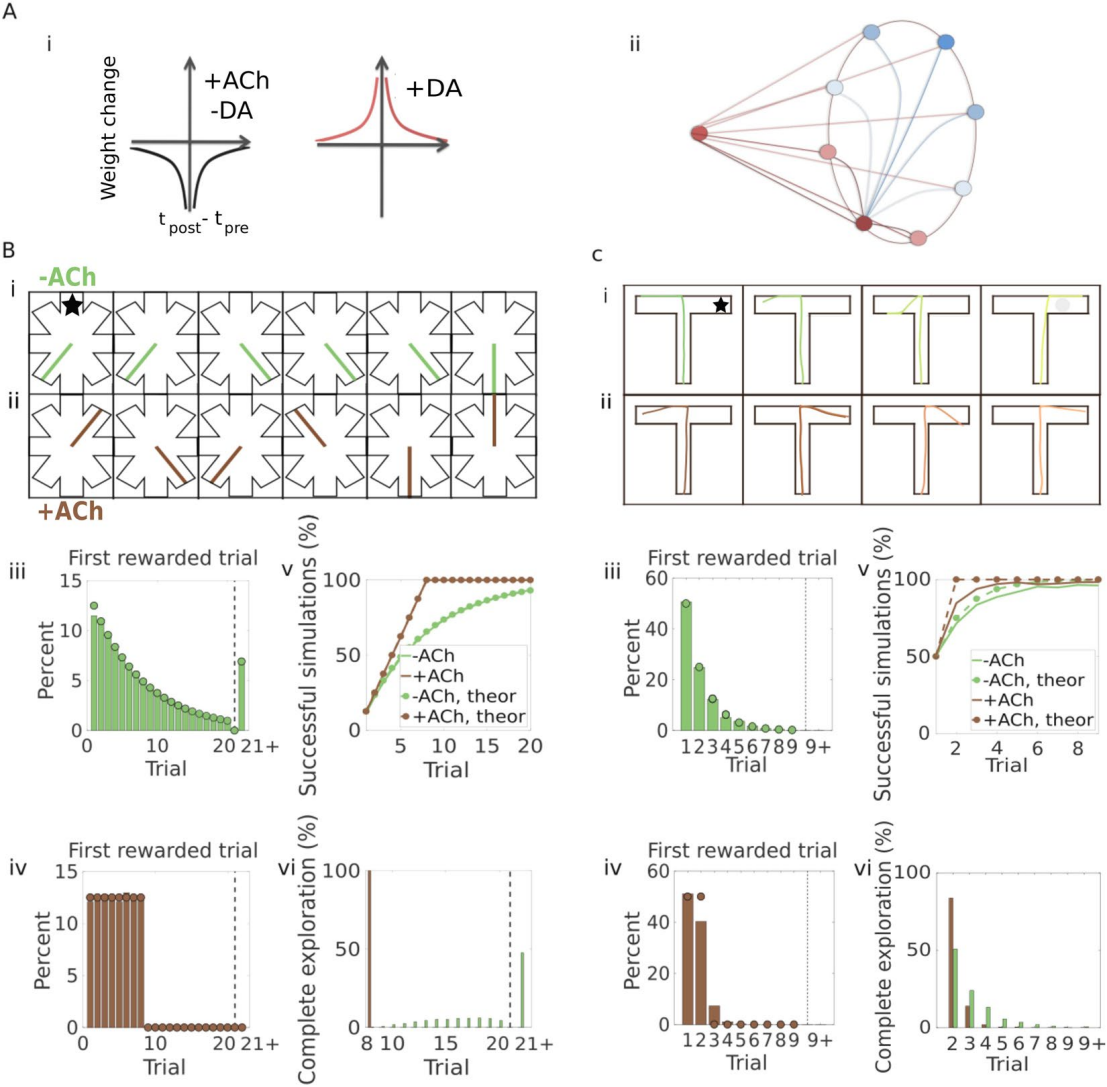
Cholinergic depression yields systematic exploration of a radial arm maze and a T-maze. (**A.i**) Sequentially neuromodulated spike-timing-dependent plasticity rule. When acetylcholine is present at the synapse, the plasticity window (black) is negative and symmetric (i.e. the weight changes proportionally to the lag between the spikes, irrespectively of the order). If dopamine is added, the plasticity window converts to positive (red). (ii) Schematic of a neural network model of a reward-driven navigation task. A place cell is connected to action neurons, which inhibit each other following a winner-take-all scheme. (**B**) Radial maze: (i-ii) Example trajectories. The maze consist of eight arms, the reward is located in the upper-central arm (with the star). (i) The agent without cholinergic depression (−ACh, green) visits the same unrewarded arm more than once. (ii) The agent with cholinergic depression (+ACh, brown) explores the maze in a systematic way: it excludes unrewarded arms and finds the rewarded arm sooner. (iii) Percent cumulative distribution of the first rewarded trial (histogram) and the corresponding theoretical distribution (geometric distribution with $p=\frac{1}{8}$; filled circles) for simulations without cholinergic depression. (iv) Percent cumulative distribution of the first rewarded trial (histogram) and the corresponding theoretical distribution (discrete uniform distribution on $[1,8]$; filled circles) for simulations with cholinergic depression. (v) Percentage of successful simulations over consecutive trials for −ACh and +ACh agents (solid lines). Theoretical learning curves, assuming one-shot learning (cumulative distribution of the first successful trial; dashed lines with dots). (vi) Agents navigating the environment without any reward. Percent cumulative distribution of the trial when the maze is fully explored (green: −ACh, brown: +ACh). (**C**) T-maze: (i–ii) Example trajectories. The maze has two arms, the reward is located in the right arm (with the star). (i) Without cholinergic interaction (−ACh), the agent consistently goes to the the same unrewarded arm. (ii) With cholinergic interaction (+ACh), the agent finds the rewarded arm sooner. (iii) Percent cumulative distribution of the first rewarded trial (histogram) and the corresponding theoretical distribution (geometric distribution with $p=\frac{1}{2}$; filled circles) for simulations without cholinergic depression. (iv) Percent cumulative distribution of the first rewarded trial (histogram) and the corresponding theoretical distribution (discrete uniform distribution on $[1,2]$; filled circles) for agents with cholinergic depression. The empirical distribution approximates the theoretical one, but does not match it exactly. (v) Percentage of successful simulations over consecutive trials for −ACh and +ACh agents (solid lines). The theoretical learning curves (assuming one-shot learning) are the cumulative distribution of the first successful trial (dashed lines with dots). (vi) Agents navigating the environment without any reward. The graph shows the percent cumulative distribution of the trial when the maze is fully explored (green: −ACh, brown: +ACh).

Based on these experimental findings, we propose a spike-timing dependent plasticity rule (Fig. 1A.i). We then explore the functional roles of our neuromodulated learning rule in a neural network (Fig. 1A.ii). Given the established role of dopamine as a reward signal and the increased release of acetylcholine during exploratory behaviours, we model navigation, specifically a task where the agent has to learn the path to the reward^32^.

### Cholinergic depression yields systematic exploration

#### Radial arm maze - discrete model

We start our investigation with a simplified network model of a radial arm maze test (Fig. 1B.i–ii). At the beginning of each trial, the agent is positioned at the centre of the maze. From there, it has to decide to which of the eight arms to move. One of the arms contains a reward (e.g. upper-central arm in Fig. 1B.i–ii), which the agent has to find and learn to reach. The network model (Fig. 1A.ii) of this task is composed by: i) a single presynaptic neuron, which can be thought of as a *place cell* coding for the position of the agent in the maze; and ii) a post-synaptic layer of eight neurons, each representing a different arm. For clarity, we call these *action neurons*, since they represent the action to take from the current position. When the trial starts and the agent is positioned in the centre of the maze, the place cell starts spiking (an inhomogeneous Poisson process, where the rate depends on the position of the agent). This, in turn, excites the action neurons (SRM_0_^33^). Due to a winner take-all connectivity in the post-synaptic layer, one of the neurons is always substantially more active by the end of the trial. The winner determines the arm that will be chosen (see Methods).

We implement our plasticity rule on the feed-forward connections between the place cell and the action neurons. Synapses follow a spike-timing dependent plasticity rule modulated by dopamine and acetylcholine. We assume that dopamine is delivered at synaptic sites whenever the agent finds the reward, and acetylcholine is present during exploration^6,10–14^ but not consummatory behaviour^4,34^. The STDP learning window is symmetric and negative under cholinergic influence (Fig. 1A.i) so, when the agent explores the environment, the active synapse gets depressed^22^. An eligibility trace (modelled as an exponential decay, see Methods) keeps track of the synaptic activity during the trial. When dopamine is delivered, synapses become potentiated retroactively by an amount proportional to the value of the eligibility trace (positive and symmetric STDP window, Fig. 1A.i). Thanks to the eligibility trace, only the most active synapse gets potentiated, and the agent can successfully learn which action leads to the reward (Fig. 1B.v)^23,24,27^. In the present task, once an arm has been chosen, there are two possible outcomes: i) the arm is rewarded, dopamine is delivered and synaptic depression is converted to potentiation, ii) the arm is not rewarded and the synapse remains depressed.

Whereas dopamine is essential to learn from a reward, acetylcholine allows learning from negative outcomes^22^. The agent is able to exclude the unrewarding arms it has already tried from future options, thereby achieving what we call *systematic exploration* (Table 1). If the effect of acetylcholine is not included in the model (−ACh), the initial exploration of the maze is entirely random. The first successful trial is thus a random variable that follows a geometric distribution with $p=\frac{1}{8}$ (Fig. 1B.iii) and mean 8. If, on the other hand, we assume perfect systematic exploration, an agent has a probability $\frac{1}{8}$ of finding the reward in the first trial, $\frac{7}{8}\frac{1}{7}=\frac{1}{8}$ in the second trial, $\frac{7}{8}\frac{6}{7}\frac{1}{6}=\frac{1}{8}$ in the third trial and so forth. The first rewarded trial is distributed as a discrete uniform random variable over the interval [1, 8] (Fig. 1B.iv, filled circles). Numerical simulations of agents with cholinergic depression (+ACh) appear to closely match the theoretical distribution (Fig. 1B.iv, histogram) which means that the agent never takes more than 8 trials to find the reward. Systematic exploration leads to the reward faster, thus enhancing the overall performance (Fig. 1B.v). Systematic exploration is further shown in a different test experiment, where we use an identical but completely unrewarded task. In this case, all the +ACh agents simulated manage to fully explore the environment by trial 8, whereas approximately half of the −ACh agents need more than 20 trials to visit all arms (*M* = 10000 simulations, Fig. 1B.vi).

**Table 1:** Exploration patterns.

	+ACh	−ACh
Radial maze	Systematic	Random
T-maze	Approximately systematic	Random
Open field	Enhanced over the action space	Random

#### T-maze - continuous model

The radial arm maze example is a very simple model of navigation that could be practically reduced to a single decision making problem. We therefore move to a more detailed, but similar model (Methods). The basic structure of the network was kept unchanged, but new features were introduced, in particular: i) infinite possible positions for the agents (inside of the maze), ii) infinite possible actions, and iii) online decision-making at each timestep.

In this task, the maze has the shape of a T (Fig. 1C.i–ii). There are *N* = 441 place cells distributed as a grid along the stem and the arms. The position of the agent is represented by a vector of its Cartesian coordinates and can be anywhere in the maze (a continuous state space). Action neurons represent each a different direction, and are arranged in a winner-take-all fashion, as before. Decisions are taken at every timestep. The direction and speed of each move is taken to be the average of the action neurons’ directions, weighted by their firing rate. This means that any arbitrary direction can be chosen (continuous action space). The recurrent connectivity ensures that only neurons with similar orientations are active at the same time. This coherent bump of activity creates smooth and consistent trajectories. The agent is limited inside the maze: if it tries to cross the boundaries, it instantly bounces back in the opposite direction (it turns by 180 degrees).

In order to test the effects of acetylcholine on systematic exploration in this model, we use a task similar to the radial maze. Each trial starts with the agent in the stem and ends when the agent enters one of the arms (or when a time limit has passed). A reward is placed in one of the arms (e.g. right arm in Fig. 1C.i–ii). The agent has to discover it and learn how to reach it.

While cholinergic depression still makes the discovery of the reward faster (Fig. 1C.i–iv) and achieves a better performance on average (Fig. 1C.v *M* = 1000 simulations), the empirical distribution does not match the theoretical distribution perfectly (~Uniform [1, 2]; Fig. 1C.iv, +ACh). When the reward is removed, full exploration of the environment is still aided by cholinergic depression. The agent fully explores the maze in just two trials in about 85% of the simulations (Fig. 1C.vi). However, exploration of the environment is more systematic with acetylcholine, but not perfect. A clearcut exclusion of wrong choices was easier to obtain in the radial maze, where there was a one-to-one correspondence between the arm and the synapse. Here in the T-maze, which has more decision points and continuous actions, more complex dynamics come into play. It is still possible to suppress wrong actions (mainly because the geometry of the maze translates into a sort of discretization of the action space), but it is difficult to achieve the same level of precision (Table 1). This concept will become clearer in the following sections, where we investigate this mechanism further by changing the maze to an open field. In an open field, no discretization is possible.

### Cholinergic depression enhances exploration of the action space

#### Learning in an open field

**Figure 2:**
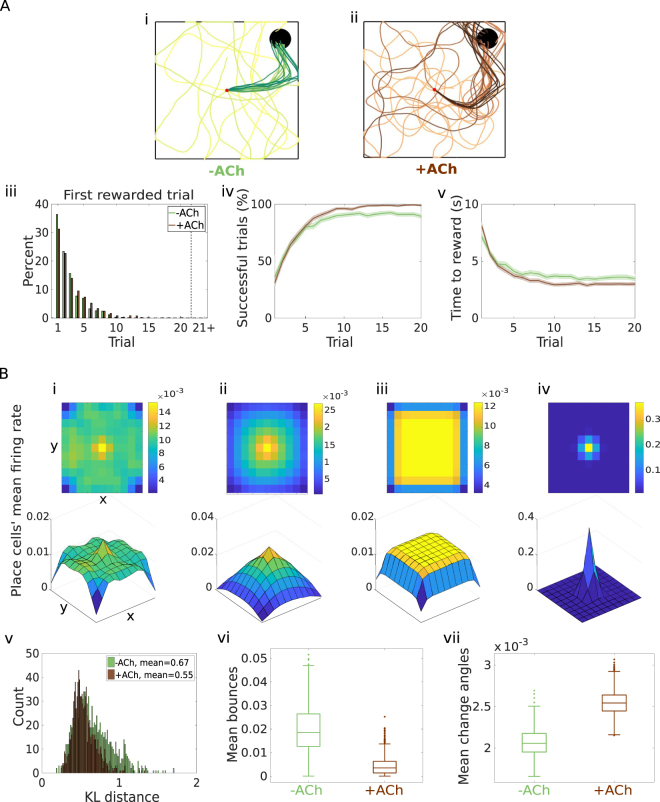
Acetylcholine-modulated plasticity enhances exploration over the action space. (**A**.i–ii) Example trajectories. Agents start each trial from the centre of the open field (red dot). Simulations without (−ACh, green) and with (+ACh, brown) cholinergic depression learn to navigate to the reward location (black circle) in 20 trials. Trials are coded from light to dark, according to their temporal order (early = light, late = dark). (iii) Reward discovery. Percent cumulative distribution of the first rewarded trial (−ACh, green histogram; +ACh, brown histogram). (iv) Learning curve presented as a percentage of successful simulations over successive trials. (v) Average time to reward in each successful trial. Unsuccessful trials, in which the agent failed to find the reward, were excluded. The shaded area (**A**.iv–v) represents the 95% confidence interval of the sample mean. (**A**.iii–v) are taken from our previous work (cf. Fig. 4A.ii–v in Brzosko *et al*.^22^). (**B**) Exploration of an open field, without any reward. (i–iv) Place cells’ activity during one trial, averaged across time (*T*_*max*_ = 15 s) and simulations (*M* = 1000), displayed over the open field; 2D (top) and 3D (bottom) views. (i) Simulations of the model without cholinergic depression (−ACh). (ii) Simulations of the model with cholinergic depression (+ACh). (iii) Benchmark simulations for Exploration over the Environment (BEE). Locations inside the field are sampled at random for the duration of one trial. (iv) Benchmark simulations for Exploration over the Action space (BEA). At each timestep, actions are chosen at random, starting from the initial position. (v) Histograms of the Kullback-Leibler divergences between each simulation (−ACh, green histogram; +ACh, brown histogram) and the benchmark simulations for exploration over the open field (Fig. 4B.iii). (vi) Boxplot of the rate of the bounces back from the walls per trial $(\frac{{\rm{n}}{\rm{o}}.\,{\rm{o}}{\rm{f}}\,{\rm{b}}{\rm{o}}{\rm{u}}{\rm{n}}{\rm{c}}{\rm{e}}{\rm{s}}}{{\rm{t}}{\rm{r}}{\rm{i}}{\rm{a}}{\rm{l}}\,{\rm{d}}{\rm{u}}{\rm{r}}{\rm{a}}{\rm{t}}{\rm{i}}{\rm{o}}{\rm{n}}})$. (vii) Boxplot of the difference between consecutive actions measured in radians. Acetylcholine yields greater variability in the action space. In a boxplot, the rectangle spans the *q*_1_ = 25th and *q*_3_ = 75th percentiles of the distribution. The line inside the rectangle is the median, and the whiskers indicate the minimum and the maximum points not considered outliers (minimum = *q*_1_ − 1.5(*q*_3_ − *q*_1_), maximum = *q*_3_ + 1.5(*q*_3_ − *q*_1_)). Points that are larger than the maximum or smaller than the minimum are outliers.

The influence of cholinergic depression on systematic exploration seems to be more complex in the continuous model. In order to study this in more detail, we choose an environment where the agent can move more freely the open field. We model the field as a square, with place cells evenly distributed over the entire area. The task is analogous to the previous ones: the agent starts each trial in the centre of the square, and has to find and learn how to reach the reward location (circle in the top right corner of the field; Fig. 2A.i–ii).

Again, due to the retroactive effect of dopamine, the vast majority of the agents are able to learn the task and navigate to the reward (Fig. 2A.iv; M = 1000 simulations under each condition) increasingly faster (Fig. 2A.v). Since dopamine affects synapses through an eligibility trace that decays over time, actions that are closer in time to reward delivery are reinforced more. Even though agents do not always pick the optimal path (Fig. 2A.i–ii), they do develop a preference for shorter paths by an amount that depends on the time constant of the eligibility trace. Thanks to cholinergic depression, +ACh agents learn to avoid unrewarding paths; this leads to increased precision in navigation and a marginal improvement in performance (Fig. 2A.iv). Unlike previous tasks, cholinergic depression does not provide any advantage in reward discovery (Fig. 2A.iii). Thus, in this particular environment, cholinergic depression does not affect systematic exploration (Table 1).

#### Exploration in an open field

We decide to further investigate the exploration patterns of our models. We remove the reward, and let the agent explore. As a proxy measure of the patterns of exploration over the open field, we take the place cells’ mean firing rates (average across time and simulations; Fig. 2B.i–iv). Once normalized to 1, place cells’ activity can be thought of as a probability distribution over the open field. This provides us with a proxy for establishing where in the field the average −ACh and +ACh agents spend the longest time. The patterns of exploration are indeed altered by cholinergic interaction: whilst +ACh agents spend more time around the centre of the field (starting position; Fig. 2B.ii) on average, −ACh agents tend to stay closer to the boundaries (Fig. 2B.i). In order to quantify the amount of exploration, we want to calculate how closely the distribution under the two conditions (−ACh and +ACh) approximates a benchmark distribution for a uniformly random exploration of the environment. The benchmark distribution was calculated by sampling (*M* = 1000) random locations inside the open field for the duration of a trial, and provides a benchmark measure for random exploration of the environment (Benchmark Exploration over the Environment, BEE; Fig. 2B.iii). We use the Kullback-Leibler divergence (KL) as a metric to quantify the difference between distributions (the more different, the higher the KL divergence). Then, we calculate the KL divergence between the distributions under either condition (−ACh and +ACh) and the benchmark (BEE). The average agent explores the environment more evenly without cholinergic interaction (KL(−ACh||BEE) = 0.03; KL(+ACh||BEE) = 0.07). However, averaging over all simulations provides only limited information about the behaviour of a single realization (as an extreme example, it could happen that each agent explores only one of the corners for the entire duration of the trial, but that it chooses one of the four corners with equal probability). We therefore also calculate the KL divergence between the output of each simulation and the benchmark (Fig. 2B.v). According to this analysis, acetylcholine seems to modestly enhance exploration. The reason for this discrepancy is that, without cholinergic depression, there is no way of suppressing unrewarding choices. Nothing prevents −ACh agents from spending a long time in the same area at the boundaries, whereas cholinergic depression encourages +ACh agents to change direction. As a consequence, −ACh agents bounce against the walls of the maze significantly more often than +ACh agents (Fig. 2B.vi).

Because of the winner-take-all connectivity of the action neurons, agents tend to keep their action choice constant and thus follow straight lines. However, acetylcholine depresses active synapses, which correspond to the currently winning action. +ACh agents are thus encouraged to pick a different action in consecutive timesteps and change direction more often (Fig. 2B.vii). This translates into more circular trajectories that tend to be focused around the centre of the maze rather than the boundaries (Fig. 2B.ii).

We can understand these findings by considering that cholinergic depression makes the postsynaptic activity (and therefore the winning action neurons) more variable, thereby enhancing exploration over the action space. This, however, does not translate into increased exploration over the open field. In order to confirm this, we use another benchmark distribution, this time as a proxy for exploration over the action space (Benchmark Exploration over the Action space, BEA). In each benchmark simulations (*M* = 1000 simulations) the position of the agent is initialized at the centre of the field. From there, every action is taken completely at random: the angle of the direction is chosen from a uniform distribution over [0, 2*π*], while the velocity is kept fixed (random walk; Methods). Place cells’ activity shows a high peak around the initial position (Fig. 2B.iv), meaning that the average benchmark agent does not move very far. As expected, the distribution of place cells’ activity in the +ACh simulations (Fig. 2B.ii) is more similar to this benchmark distribution than −ACh simulations (KL(−ACh||BEA) = 13.12, Fig. 2B.i; KL(+ACh||BEA) = 9.7, Fig. 2B.ii). In conclusion, acetylcholine enhances exploration over the action space but not necessarily over the environment (Table 1).

### Cholinergic depression improves performance in dynamic environments

#### Relearning in an open field

**Figure 3:**
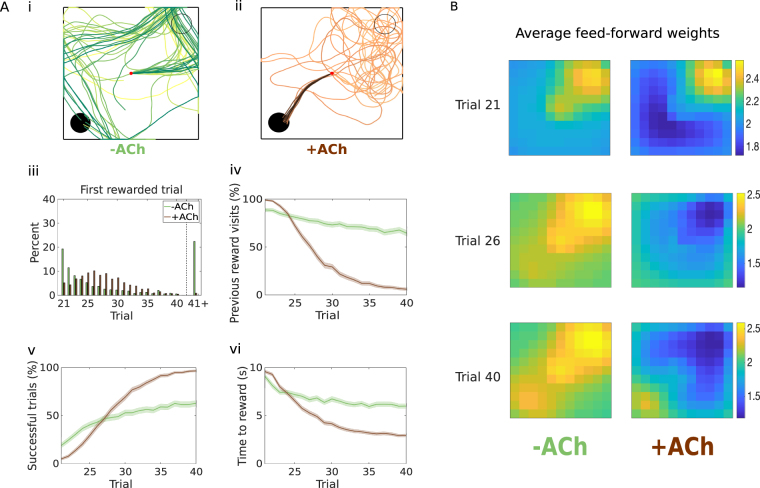
Acetylcholine improves performance in dynamic environments. (**A**) After the 20 initial trials (Fig. 2A), the reward is moved to the opposite corner of the open field (old location = hollow black circle, new location = solid black circle). Trials are coded from light to dark, according to their temporal order (early = light, late = dark; Trials 21–40). (i) Agents without cholinergic depression do not unlearn the old path, but they can extend it to the new rewarded location. (ii) Agents with cholinergic depression can unlearn a previously learned path. (iii) Reward discovery. Percent cumulative distribution of trials when the reward is discovered for the first time. (iv) Percentage of agents visiting the old reward location as a function of the trial index. (v) Percentage of successful simulations as a function of the trial number. (vi) Average time to reach the new reward (only successful trials). The shaded area (**A**.iv–vi) represents the 95% confidence interval of the sample mean. (**A**.iii–vi) are taken from our previous work (cf. Fig. 4B.ii–v in in Brzosko *et al*.^22^). (**B**) Feed-forward weights, averaged across action neurons, displayed as an image over the open field. Each square represents the mean synaptic weight connecting the place cell centered in that location to all action neurons. Synaptic weights were stored at the beginning of the trials (21, 26, 40) and are averaged across *M* = 1000 simulations.

We have shown that cholinergic depression allows the agent to learn from negative outcomes and increases exploration over the action space. These characteristics suggest that cholinergic depression might be especially advantageous in dynamic environments. We consider a task in which, after 20 initial trials where the agent learns how to navigate to the reward (Fig. 2A), the reward is moved to a new location. In our case, it is moved to the opposite corner (Fig. 3A.i–ii). +ACh agents discover the new reward location in fewer trials, while as much as one out of four −ACh agents cannot find it before the end of the experiment (Fig. 3A.iii)^22^. In addition, the +ACh agents show better task performance than the −ACh agents (96.8% correct versus 63% correct; Fig. 3A.v). −ACh agents mostly just extend the previously learned path (Fig. 3A.i), whereas +ACh agents stop visiting the old reward location altogether (Fig. 3A.ii,iv). This results in a difference in the time to navigate to the new reward location (Fig. 3A.vi). Even with the addition of noise in the neural activity (Supplementary Fig. 1) and in the weights (Supplementary Fig. 2), −ACh cannot achieve the same degree of behavioural flexibility (Table 2).

**Table 2:** Behavioural flexibility.

	+ACh	−ACh	r-STDP	Dynamic reward	Negative feedback
learning	Yes	Yes	If STDP integral > 0	Mostly (better for small *β*)	Yes
unlearning	Yes	No	If STDP integral < 0	Partially (better for large *β*)	Partially

There are two main mechanisms underlying the behavioural flexibility exhibited by +ACh (Fig. 3B). On one hand, cholinergic depression decreases the strength of synapses associated to those actions that are no longer rewarding (Trial 26, lower weights in the upper-right corner). On the other hand, retroactive dopaminergic potentiation allows the agent to learn new sequences of actions that lead to the reward (Trial 40, higher weights in the bottom-left corner). Behaviourally, this results into the extinction of the previously learned path and the acquisition of a newly rewarding path. Thanks to dopaminergic potentiation, −ACh agents can also learn the path to the new reward location (Trial 26 and 40, weights in the bottom-left corner are higher than average), but they cannot unlearn the old reward location, which remains the agents’ most followed path (highest weights in the top-right corner; Fig. 3A.iv). It is worth noting here that acetylcholine affects all synapses, not only the ones that had been previously potentiated. For example, in the first part of the experiment, +ACh agents learn to navigate to the reward (Trial 21, higher weights in the top-right corner) but also to avoid unrewarding paths (Trial 21, lower weights in the left-bottom corner). This increases the precision of +ACh agents and improves performance (Fig. 2A.iv). For this reason, we use here the generic term “unlearning” to indicate the effect of synaptic depression on any sequence of actions that becomes less likely to be chosen again.

#### Learning and relearning in an open field with obstacles

**Figure 4:**
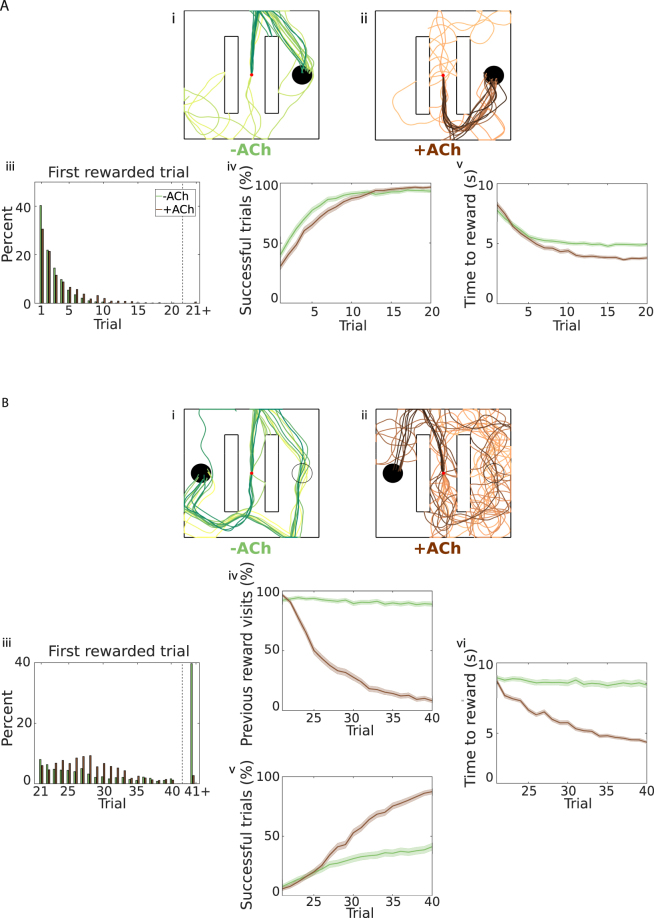
Flexible learning in an open field with obstacles. (**A**) Trials 1–20. Agents start each trial from the centre of the open field (red dot) and have to avoid the obstacles (white bars). Agents with and without cholinergic depression (+ACh and −ACh respectively) learn to navigate to the reward location (black circle). (i-ii) Example trajectories. Trials are coded from light to dark, according to their temporal order (early = light, late = dark). (iii) Reward discovery. Percent cumulative distribution of the first rewarded trial. (iv) Percentage of successful simulations across trials. (v) Average time to reward in each trial (only successful simulations). (**B**) Trials 21–40. The reward is moved to the opposite side of the open field. Agents with and without cholinergic depression (+ACh and −ACh respectively) learn to navigate to the new reward location (solid black circle). (i) Example trajectories. Without cholinergic depression, the agent learns a route to the new reward location, but mainly as an extension of the path learned previously. (ii) The agent with cholinergic depression unlearns the path to the previous reward location (hollow black circle) and navigates to the new reward. (iii) Reward discovery. Percent cumulative distribution of trials when the reward is discovered for the first time. (iv) Percentage of agents visiting the old reward. (v) Percentage of successful simulations as a function of the trial number. (vi) Average time to reach the new reward (only successful trials). The shaded area (**A**.iv–v and **B**.iv–vi) represents the 95% confidence interval of the sample mean.

As mentioned earlier, the specifics of the task strongly affect the outcome. To explore this point further, we repeat the same experiment using a slightly different maze geometry. We insert two vertical obstacles in the open field, and move the reward location on the x axis (*y* = 0), to the right side of the obstacles, for the first part of the experiment (Fig. 4A.i–ii; obstacles = white vertical bars, reward location = black solid circle). In this case, −ACh agents initially perform better at finding the reward: 40% find the reward in the first trial, in contrast to just 30% of +ACh agents (Fig. 4A.iii). It is much easier to discover the reward when following straight lines in this particular maze geometry (even more so than in a simple open field; Fig. 4A.i–ii). Later in the experiment (Trial 20), however, agents equipped with cholinergic depression achieve a slightly higher success rate (Fig. 4A.iv), and are faster to navigate to the reward (because they do not get stuck against the walls or the obstacles; Fig. 4A.i–ii,v). The results for the second part of the experiment, when the reward is moved horizontally to the left side of the obstacles, are qualitatively similar to the open field but even more pronounced (Fig. 4B). Almost 40% of −ACh agents (39.7%) do not find the new reward before the end of the experiment (Fig. 4B.iii), and 89.2% of them still visit the old reward location in the last trial (Fig. 4B.iv). With this maze geometry, it is more difficult to extend the old path to the new reward location. More prominently than in the open field, agents with cholinergic depression are twice as successful as −ACh agents (Fig. 4B.v) and can navigate to the reward twice as fast (Fig. 4B.vi).

### Comparison with other learning rules

#### Reward-modulated STDP

Until now, we have focused on the functional role of cholinergic depression, comparing the same learning rule with and without cholinergic depression (+ACh and −ACh). We next investigate how sn-Plast compares to other reward-modulated learning rules.

**Figure 5:**
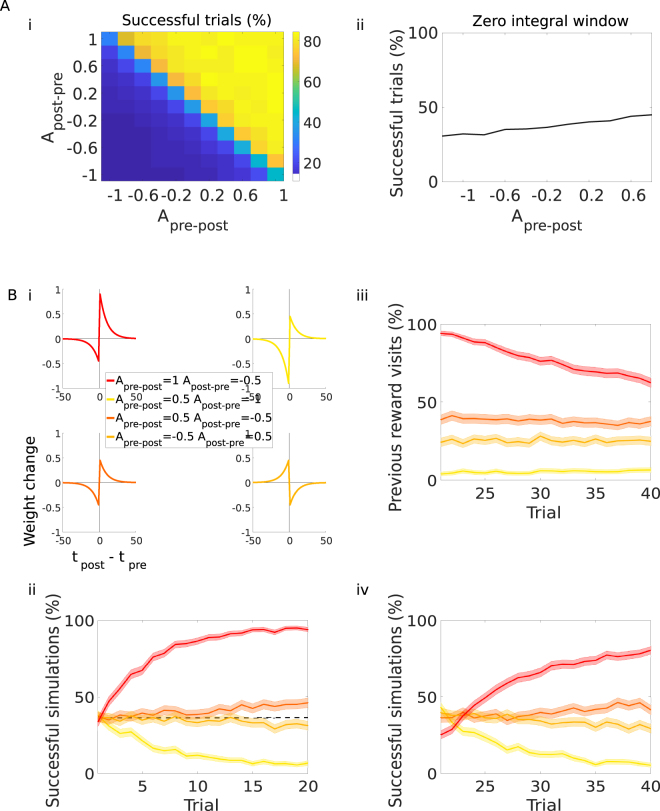
Comparison with reward-modulated STDP. Agents equipped with reward-modulated STDP with varying parameters *A*_*pre*–*post*_ and *A*_*post*–*pre*_ learn to navigate towards a reward in an open field. (**A**) Parameters sweep. We run *M* = 200 simulations of the task in Fig. 2A (learning to navigate to the reward in the top-right corner of the open field, 20 trials). (i) Percentage of successful trials, as a function of the amplitudes of the plasticity windows (pre-post and post-pre). The integral of the STDP window mostly determines the agent’s performance. (ii) Percentage of total successful trials for an STDP window with vanishing integral (*A*_*pre*–*post*_ + *A*_*post*–*pre*_ = 0, diagonal of the matrix). (**B**) Simulations of the dynamic task, as in Figs 2A and 3, for four different parameters sets. (i) Legend and representation of the four learning windows: positive integral (red), negative integral (yellow), zero integral with *A*_*pre*–*post*_ > 0 (dark orange) and zero integral with *A*_*pre*–*post*_ < 0 (light orange). (ii) Agents have to learn to navigate to the reward in the top-right corner of the open field, trials 1–20. Percentage of successful simulations. The dashed line indicates the baseline performance. (iii-iv) The reward is moved to the opposite corner of the open field, trials 21–40. (iii) Percentage of agents visiting the previously rewarded location. (iv) Percentage of successful simulations. The shaded area (**B**.ii–iv) represents the 95% confidence interval of the sample mean.

To this end, we change the plasticity rule in our model to standard reward-modulated STDP (r-STDP; Fig. 5). In r-STDP, synapses follow a classical STDP rule with different amplitudes for the pre-post (*A*_*pre*–*post*_) and post-pre (*A*_*post*–*pre*_) windows (e.g. Fig. 5B.i). However, all synaptic changes are gated by dopamine and become effective only retroactively through an eligibility trace. If no reward is found, weights are left unchanged. Notably, if the amplitudes of the r-STDP learning window are set to *A*_*pre*–*post*_ = *A*_*post*–*pre*_ = +1, reward-modulated STDP is equivalent to the plasticity rule used in our control simulations (sn-Plast without acetylcholine; −ACh).

We then investigate how the agent performs when equipped with r-STDP. We start from testing learning in a static environment. As before, the agent moves in an open field and has 20 trials to learn to navigate to the reward location (Fig. 2A). We then run a parameters sweep, and examine how the agent’s performance (mean percentage of successful trials, *M* = 200 simulations; Fig. 5A.i) varies with the amplitudes of the learning window (*A*_*pre*–*post*_ and *A*_*post*–*pre*_). When there is no learning (*A*_*pre*–*post*_ = *A*_*post*–*pre*_ = 0; middle square, Fig. 5A.i), the average performance is 37%. Using this value as baseline, we can determine whether agents learn or unlearn. The agents’ performance varies as a function of the integral of the learning window: it rises above baseline for positive-integral windows (*A*_*pre*–*post*_ + *A*_*post*–*pre*_ > 0; the part above the diagonal, Fig. 5A.i) and below baseline for negative-integral windows (*A*_*pre*–*post*_ + *A*_*post*–*pre*_ < 0; the part under the diagonal, Fig. 5A.i). When the integral of the plasticity window is zero (*A*_*pre*–*post*_ + *A*_*post*–*pre*_ = 0; diagonal of the matrix, Fig. 5A.i), there is little variation from baseline. However, the performance clearly increases with the amplitude of the pre-post learning window, *A*_*pre*–*post*_ (Fig. 5A.ii). This is because, in a spiking neural network, presynaptic spikes contribute to elicit postsynaptic spikes (spike-spike correlation^35^). As such, the amplitude of the pre-post window, relatively to the post-pre window, brings an extra contribution to learning. We can conclude that the order of the spikes matters, although only marginally so. In our model, what really determines whether the agent learns or unlearns is the integral of the STDP window^31^.

We next compare four agents, equipped with different STDP windows having: i) positive integral (red), ii) negative integral (yellow), iii) zero integral and *A*_*pre*–*post*_ > *A*_*post*–*pre*_ (dark orange) and iv) zero integral and *A*_*pre*–*post*_ < *A*_*post*–*pre*_ (inverse STDP window; light orange). As expected, the best learner is the agent with the positive learning window, whereas the agent with a negative learning window effectively unlearns (Fig. 5B.ii). There is generally very little change in performance when the integral of the STDP window is zero: if *A*_*pre*–*post*_ > *A*_*post*–*pre*_, we can observe some slow learning; if *A*_*pre*–*post*_ < *A*_*post*–*pre*_ there is very slow unlearning instead (Fig. 5B.ii). As mentioned earlier (Fig. 5A.ii), the spike order is still relevant, although only marginally so. This is due to spike-spike correlation^35^. These patterns remain consistent in the second part of the experiment, when the reward is moved to a different corner of the field. The agent with a positive integral learns how to navigate to the new reward location (Fig. 5B.iv) but does not really unlearn the path to the first reward (the visits to the previously rewarded location are still as high as 62.4% at trial 40; Fig. 5B.iii). The agent with a negative integral completely unlearns the path to the second reward too (Fig. 5B.iv). Agents with vanishing integrals show very little change in both learning of the new reward location and unlearning of the old one (Fig. 5B.iii–iv).

Thus, r-STDP allows the agent to either learn or unlearn the path to the reward, depending on the integral of the learning window. This learning rule, however, appears to be quite rigid. It lacks the flexibility of sn-Plast which, thanks to the modulation of both acetylcholine and dopamine, can switch between these modalities in response to environmental changes (Table 2). For this reason, sn-Plast is more suited to dynamic tasks that require a degree of adaptation. This analysis also shows how the spike order is only relatively important to learning. This characteristic is intrinsic to the model^31,32^ and in striking agreement with the experimental data from which we derive our plasticity rule (both dopaminergic and cholinergic modulated STDP windows are symmetric and therefore invariant to spike order^21,22^).

#### Dynamic reward signal

In sn-Plast, the reward signal is binary, it is either present or not. Alternatively, we could conceive signals with more complex temporal dynamics. In particular, we want to focus here on a signal that keeps track of the history of the reward delivery. This is particularly useful in a changing environment and worth comparing to our sequentially neuromodulated plasticity rule. We employ a dynamic reward signal, *ρ*(*tr*), given by the difference between the raw reward, *R*(*tr*), and a moving average of the past rewards, $\bar{R}(tr)$: $\rho (tr)=R(tr)-\bar{R}(tr)$ (Methods). Synapses modulated by the dynamic reward signal *ρ*(*tr*) are updated only if the outcome of trial *tr* is somewhat surprising, that is, if it differs from the outcomes of the most recent trials. The effect is twofold: if the reward is reached consistently and continuously, the average reward becomes very close to the actual reward value, *ρ*(*tr*) ≈ 0, and synapses stop being potentiated; if the agent stops receiving a reward suddenly ($R(tr) < \bar{R}(tr)$, second half of the experiment), the dynamic reward signal becomes negative and synapses are depressed.

**Figure 6:**
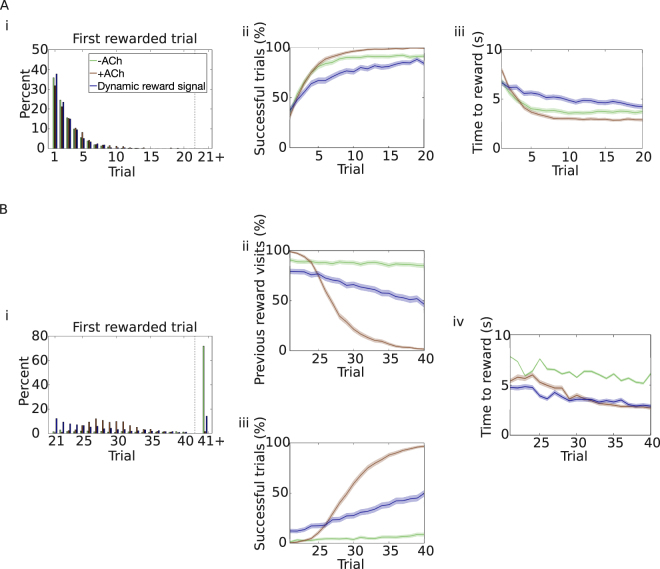
Comparison with dynamic reward signal. (**A**) Trials 1–20. Agents start each trial from the centre of the open field and have to navigate to the top-right corner (task as in Fig. 2A). Simulations are run under three different conditions: with only dopaminergic potentiation (−ACh, green); with dopaminergic potentiation and cholinergic depression (+ACh, brown); with STDP modulated by a dynamic reward signal (blue). (i) Reward discovery. Percent cumulative distribution of trials when the reward is discovered for the first time. (ii) Percentage of successful simulations across trials. (iii) Average time to reward in each trial (only successful simulations). (**B**) Trials 21–40. The reward is moved to the opposite side of the open field (task as in Fig. 3A; but the task is stopped whenever the agent enters either the new or the old rewarded area). (i) Reward discovery. Percent cumulative distribution of trials when the reward is discovered for the first time. (ii) Percentage of agents visiting the old reward location. (iii) Percentage of successful simulations as a function of the trial number. Acetylcholine yields the best performance. (v) Average time to reach the new reward (only successful trials). The shaded area (**A**.ii–iii and **B**.ii–iv) represents the 95% confidence interval of the sample mean.

In order to test the performance of this different learning rule, we use a similar task as before. In the first half of the experiment, the agent moves in an open field and has to learn how to navigate to the reward (as in the previous task, Fig. 2A). Agents receiving a dynamic reward signal are disadvantaged in this initial learning phase. Even though they are equally fast to discover the reward (Fig. 6A.i), they are slower to learn how to reach the reward location, and they are less successful (Fig. 6A.ii). Even when they learn the path to the reward, they take longer to reach it (Fig. 6A.iii). In general, learning is less efficient when using a dynamic reward signal. In the second part of the experiment, from trial 21 to trial 40, the reward is moved to the opposite corner of the environment. Unlike before, however, a trial ends if the agent enters either one of the rewarding areas (old or new), or if a time limit is reached. The agent has to discover the novel reward location and learn a new path. Agents equipped with the dynamic reward signal plasticity rule outperform −ACh agents, but their performance is still inferior to +ACh agents. Only 14% of the dynamic reward signal agents do not manage to discover the reward by the end of the experiment, this is significantly better than our control simulations (−ACh, 71.9%) but worse than sn-Plast agents (+ACh, 1.6%; Fig. 6B.i). Notably, the dynamic signal allows for some unlearning of the previous reward location (Fig. 6B.ii). Approximately half of the agents can reach the novel reward location by the end of the experiment (Fig. 6B.iii), and can do so fairly quickly (Fig. 6B.iv) suggesting that they do indeed learn a completely new path. However, at least 50% of the agents still visit the old reward location by trial 40, as opposed to almost zero +ACh agents (Fig. 6B.iii). Overall, agents equipped with sn-Plast outperform agents receiving a dynamic reward signal.

The dynamic reward signal can be both positive and negative in sign, this allows for both learning and unlearning of the appropriate actions. Even though this rule provides more flexibility than classic reward-modulated STDP, the mechanisms for learning and unlearning are still highly connected. Weight changes are regulated by the timescale of integration of the moving average reward (Methods). In the first half of the experiment, a longer timescale leads to improved performance: the average reward $\bar{R}$ requires more successful trials to converge to *R*, so synapses get potentiated more (Supplementary Fig. 3A). However, a longer timescale also implies that if the reward is moved, it will take longer to depress the appropriate synapses and unlearn the unrewarding path (Supplementary Fig. 3B). The dynamic reward signal compares poorly with our sequentially neuromodulated rule: sn-Plast uses separate mechanisms to learn and unlearn, increasing flexibility and improving performance (Table 2).

#### Negative feedback

In sn-Plast, synapses are biased towards depression unless a reward is delivered. This kind of depression allows suppression of a previously learned sequence of actions, but it is indiscriminately persistent throughout exploration and is not specific to reward omission. Alternatively, we could imagine that a negative feedback is delivered to the synapse when the expected reward is omitted. This negative signal would retroactively depress synapses through the use of an eligibility trace, similarly to dopamine but opposite in sign. The synaptic change would then be positive if the reward is delivered (*A*_*feedback*_ = 1) and negative if it is omitted (*A*_*feedback*_ = −1). This feedback signal is reminiscent of a prediction error^1^, but different in that the expectations are not updated during the experiment. We thus compare sn-Plast to this model with targeted negative feedback. As before, the agent explores the open field for the first 20 trials and has to learn how to navigate to the reward. For the remaining 20 trials the reward is moved to the opposite corner of the field, and the agent has to discover it and learn the new path. Unlike before, however, a trial ends if the agent enters either one of the rewarding areas (old or new), or if a time limit is reached. Whenever the agent enters the old reward location, a negative feedback signal induces synaptic depression.

**Figure 7:**
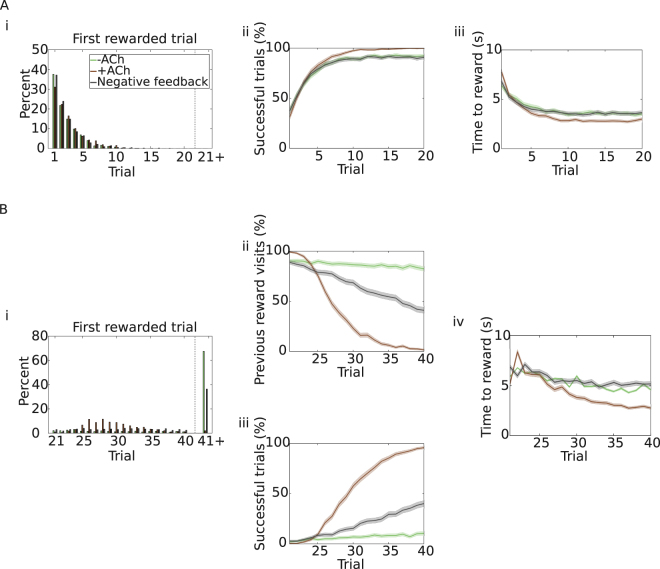
Comparison with learning from negative feedback. (**A**) Trials 1–20. Agents start each trial from the centre of the open field and have to navigate to the top-right corner (task as in Fig. 2A). Simulations are run under three different conditions: with only dopaminergic potentiation (−ACh, green); with dopaminergic potentiation and cholinergic depression (+ACh, brown); with dopaminergic potentiation and negative feedback (grey). (i) Reward discovery. Percent cumulative distribution of trials when the reward is discovered for the first time. (ii) Percentage of successful simulations across trials. iii) Average time to reward in each trial (only successful simulations). (**B**) Trials 21–40. The reward is moved to the opposite side of the open field (task as in Fig. 3A; but the task is stopped whenever the agent enters either the new or the old rewarded area). (i) Reward discovery. Percent cumulative distribution of trials when the reward is discovered for the first time. (ii) Percentage of agents visiting the old reward location. (iii) Percentage of successful simulations as a function of the trial number. Acetylcholine yields the best performance. (v) Average time to reach the new reward (only successful trials). The shaded area (**A**.ii–iii and **B**.ii–iv) represents the 95% confidence interval of the sample mean.

Since no negative feedback is present in the first half of the task, agents with negative feedback signal perform identically to −ACh agents (Fig. 7A). The continuous updating of cholinergic depression increases the success rate (Fig. 7A.ii) and diminishes the average time to reward (Fig. 7A.iii), but it slightly deteriorates the initial exploration (it takes longer to discover the reward; Fig. 7A.i). It is in the second part of the experiment, after reward displacement, that we see the effect of the negative feedback. As expected, −ACh agents show the poorest performance (Fig. 7B). In contrast, agents with negative feedback signal are able to partially unlearn the previously rewarded location (Fig. 7B.ii). They can therefore also find (Fig. 7B.i) and reach (Fig. 7B.iii) the newly rewarded location more often. Nevertheless, +ACh agents still show the best results. They unlearn the old reward location completely (Fig. 7B.ii). They also find and learn the new reward location, reaching an almost perfect performance (Fig. 7B.iii). The time to the reward is also shorter for +ACh agents, whereas almost no difference can be found between the other two sets of simulations (Fig. 7B.iv).

Targeted negative feedback acts retroactively through an eligibility trace. Consequently, synapses active more recently get depressed more. This mechanism allows suppression of previously rewarded actions to some extent, but performs quite poorly when compared to cholinergic depression, at least in the current model (Table 2). Cholinergic depression acts equally on all synapses throughout exploration, and therefore offers a more powerful and direct way of unlearning.

## Discussion

In this paper we investigated the possible functional consequences of neuromodulated hippocampal STDP, based on our recent experimental findings^22^. In particular, we analyzed this plasticity rule in a network model of reward-driven navigation. Consistent with previous models, dopamine makes it possible to learn the path to the reward^23,24,27^. Acetylcholine, instead, allows learning from negative outcomes. This yields behavioural flexibility and is particularly useful in dynamic environments, where it is necessary to both learn and unlearn in a task-relevant manner. In a simple model with discrete state and action space, cholinergic depression allows suppression of unrewarding choices and systematic exploration of the maze. In more complex continuous models, it enhances exploration over the action space, but this does not necessarily translate into increased exploration over the entire maze.

### Dopamine

Dopamine is thought to signal reward delivery and reinforce behaviour^1–3^. The behavioural^36–38^ and algorithmic^39^ mechanisms of reward-modulated learning have been thoroughly investigated and characterized. Recently, its neural substrates have been explored as well. Dopamine has been reported to shift STDP towards potentiation^18–20^. In this study, we build on our previous experimental findings on the retroactive effect of dopamine on hippocampal synaptic plasticity^21^. Taking inspiration from both reinforcement learning^39^ and biology^40^, dopamine was theorized to act on synaptic eligibility traces. These traces keep track of and reinforce neural activity associated with distal rewards^23,24,27^. Given the similarity of sn-Plast to other reward-modulated plasticity rules^23^, our network unsurprisingly succeeds in learning rewarded patterns of activity. Nevertheless, our learning rule does differentiate itself from other reward-modulated plasticity rules because of the symmetrical positive shape of its STDP window. Consistently, in our model the integral of the learning window has greater importance than the exact spike timing^35^. In a different framework, however, spike timing could have functional roles, for instance when precise spike sequences are learned^23,24,31^.

One limitation of our model is that we assume that dopamine signals exclusively reward. As such, it would only update synaptic weights during reward delivery. However, dopamine has also been associated with spatial novelty in the hippocampus and has been shown to correlate with exploratory behaviour^41–46^. In fact, the role of dopamine as a reward signal in the hippocampus has been challenged because of the sparsity of the projections from VTA^44,47^. Nevertheless, more recent research points towards a role for dopamine in reinforcing spatial representations in the hippocampus^41^ and goal-directed navigation^47,48^.

We approximate dopamine as a stable reward signal that is available globally at the synapses with every reward delivery. However, dopaminergic neurons exhibit different modes of firing, with phasic firing coding for reward prediction error. As such, dopamine is released only when the reward is unexpected^1–3^. If the animal was able to predict the reward delivery correctly, then VTA dopaminergic neurons would not increase their firing rate.

In our work, we compared sn-Plast to other plasticity rules. In particular, we explored: i) a dynamic reward signal, which carries information about the history of the past trials, and ii) a negative feedback signal, which is released selectively when the agent enters a previously rewarded location. Although clearly different, these signals are reminiscent of a prediction error. The dynamic reward signal favours synaptic updates that are “surprising” and it can be both positive and negative in sign. The negative feedback signal is completely symmetric and opposite in sign to the reward signal, as such, it constitutes a limit case of the reward prediction error (the prediction error can only be larger or equal to the negative feedback signal). We showed that sn-Plast outperforms both plasticity rules. This is because cholinergic depression acts as a more general and separate mechanism from dopaminergic potentiation (i.e. it acts on synapses immediately, not through an eligibility trace, and acts indiscriminately on all unrewarding synapses). We could speculate that, for analogous reasons, sn-Plast would similarly outperfom a prediction error signal.

Frémaux *et al*. proposed a spiking implementation of the prediction error in a similar reward-driven network model^32^. The agents were able to perform very complex tasks but, unfortunately, the authors did not investigate changing environments. Although we imagine that sn-Plast would outperform a prediction error-based learning rule, it could be interesting to substitute the reward signal in sn-Plast with a prediction error. Cholinergic depression might interact with the prediction error dynamics, probably affecting both exploration and performance. Interestingly, in Frémaux *et al*.^32^ no backpropagation of the prediction error was observed. This suggests that the dynamics of the prediction error in this specific framework might be different or unexpected.

### Acetylcholine

Acetylcholine is known to play an important role in learning and memory^49–51^. In the hippocampus, acetylcholine has been reported to facilitate both long term potentiation^52–62^ and long-term depression^63–67^, depending on a number of variables, such as plasticity induction protocols, acetylcholine concentrations and type of cholinergic receptors^28^. In our previous work^22^, we found that cholinergic modulation of hippocampal STDP resulted in a symmetrical negative learning window and used this data as a starting point for our investigation.

Acetylcholine has been studied in relation to behavioural tasks^50,68,69^. Microdialysis studies have reported an increase in cholinergic release in the hippocampus during engagement in spatial learning tasks^6,8,10–14^ and a reduction during consummatory behaviour^4,34^. Adding this dynamic to our neuromodulated network allowed us to study the possible effects of acetylcholine on navigation and decision-making^22^. Acetylcholine has been postulated to signal novelty and saliency^6,7,9^, and was reported to enhance exploratory behaviours like rearing^8,70^. For this reason, we largely focused on characterizing the effect of acetylcholine on exploration. In our model, acetylcholine indeed increases exploration over the action space, but this does not necessarily translate into increased exploration over the entire physical environment. In addition to exploration, the effect of acetylcholine on spatial learning has also been connected to paradigm shifts and reversal learning^71–73^, which in turn has been shown to depend on long-term depression in hippocampal synapses^74^. This is in agreement with our observation that acetylcholine is useful in dynamic scenarios where unlearning previously learned actions is advantageous.

Acetylcholine has been hypothesized to modulate learning in other computational theories before^75–77^. It was put forward as a signal for uncertainty in probabilistic environments^76^ and a switch signal for the encoding of new information, as opposed to the consolidation of memories^75,77^. Finding a clear correspondence between these theories and the model we present here is not trivial. However, our results are consistent with previous work, in that they suggest a functional role for acetylcholine in learning which is: i) complementary to dopamine, and ii) relevant to dynamical, changing environments.

## Conclusion

In conclusion, we model here a role for dopamine as a behavioural reinforcer, and propose a new role for cholinergic depression in learning from negative outcomes. Despite its simplicity, our feed-forward network captures the key characteristics of sequentially neuromodulated plasticity, allowing us to examine its potential role in reward-based navigation^22^. In addition, by allowing us to clearly examine its dynamics, it provides us with a useful tool to further investigate the relationship between synaptic and behavioural learning. The continuosly updated cholinergic depression allows learning from unsuccessful trials, unlearning of previously rewarded locations, and enhances exploration over the appropriate action space. As such, sn-Plast is an effective reward-modulated learning rule for navigation tasks.

## Methods

The navigation model is based on a one-layer network^32^. The *place cells* in the input layer code for the position of the agent in the environment. They project to the output layer of *action neurons*. Each one of the action neurons represents a different direction. Lateral connectivity in this layer ensures that action neurons compete with each other in a winner-take-all scheme. Their activity is then used to determine the action (i.e. direction and velocity) to take at every instant.

### Place cells

#### Discrete model

In the case of the radial maze, the state space is discrete and contains only one location: the centre of the maze. From there, the agent chooses to which of the eight possible arms to move. The network is therefore composed of a single place cell, active for the whole duration of the trial, simulated as a Poisson process with rate ${\bar{\lambda }}^{pc}=4000$ Hz.

#### Continuous model

The position of the agent at time t is described by the two-dimensional vector of its Cartesian coordinates, **x**(*t*). There are *N* place cells, spread over the entire environment at a horizontal and vertical distance of *σ* from one another. The spiking activity of place cell *i* is modelled as an inhomogeneous Poisson process, with rate ${\lambda }_{i}^{pc}({\bf{x}}(t))$ defined as follows:

1 $${\lambda }_{i}^{pc}({\bf{x}}(t))={\bar{\lambda }}^{pc}\,\exp (-\frac{\parallel {\bf{x}}(t)-{{\bf{x}}}_{i}{\parallel }^{2}}{{\sigma }^{2}}).$$

The firing rate ${\lambda }_{i}^{pc}$ is a function of the distance of the agent from the place cell centre *x*_*i*_. It is at its maximum, ${\bar{\lambda }}^{pc}=400$ Hz, when the agent is located exactly in **x**_*i*_ and it decreases as it moves away. This mechanism simulates a place field in a 2D environment, which allows for an accurate representation of the position of the agent in the environment. In both models the firing rates of the place cells are taken to be very high, this is just to speed up computational times while preserving navigation accuracy.

Open field: The open field is modelled as a square of side length of 4 a.u. The initial position of the agent in each trial is the centre of the open field, which corresponds to the origin of the Cartesian plane.When obstacles are added, they are modelled as two rectangular bars of sides *sx*_*obs*_ = 0.4 a.u. and *sy*_*obs*_ = 0.8 a.u. centred on the x axis at *x*_1_ = −1.2 a.u. and *x*_2_ = 1.2 a.u. In the open field, there are *N* = 121 place cells at distance *σ* = 0.4 from one another.

T-maze - continuous model: The T-maze is cropped out from the open field plane. It is composed by a stem of length *l*_*stem*_ = 3.2 a.u. and width *wd*_*stem*_ = 0.6 a.u., and two arms, each having length *l*_*arm*_ = 1.7 a.u. and width *wd*_*arm*_ = 0.8 a.u. The agent starts every trial from the bottom of the stem: (*x*_*start*_, *y*_*start*_) = (0, −2) a.u. In the T-maze, there are *N* = 441 place cells at distance *σ* = 0.2 from one another.

### Action neurons

#### Neuron model

Place cells constitute the input to the network, and they all project to all action neurons with weights *w*^*feed*^. These feed-forward weights are initialized to *w*_*in*_ and bounded between *w*_*min*_ and *w*_*max*_ (see Table 3 for specific values). The feedforward weights should be initialized roughly halfway between the minimum and the maximum value, so that both cholinergic depression and dopaminergic potentiation can have an effect on the action choice. Action neurons are also connected with each other through synaptic weights *w*^*lat*^. The neurons are modelled as SRM_0_^33^, the membrane potential of neuron *j* is therefore given by:

2 $${u}_{j}(t)=\sum _{i}\,\sum _{{\bar{t}}_{i}\in {F}_{i}^{pc},t > {\hat{t}}_{j}}\,{w}_{ji}^{feed}\cdot \varepsilon (t-{\bar{t}}_{i})+\sum _{k,k\ne j}\,\sum _{{\bar{t}}_{k}\in {F}_{k}^{a},t > {\hat{t}}_{j}}\,{w}_{jk}^{lat}\cdot \varepsilon (t-{\bar{t}}_{k})+\chi {\rm{\Theta }}(t-{\hat{t}}_{j})\,\exp (-\frac{t-{\hat{t}}_{j}}{{\tau }_{m}}),$$

where *χ* = −5 mV scales the refractory period, ${\hat{t}}_{j}$ is the last postsynaptic spiking time and *ε* is the EPSP described by the kernel $\varepsilon (t)=\frac{{\varepsilon }_{0}}{{\tau }_{m}-{\tau }_{s}}({e}^{\frac{-t}{{\tau }_{m}}}-{e}^{\frac{-t}{{\tau }_{s}}}){\rm{\Theta }}(t)$, with Θ(*t*) being the Heaviside step function, *τ*_*m*_ = 20 ms, *τ*_*s*_ = 5 ms, $${\epsilon }_{0}=\{\begin{array}{cc}10 & {\rm{f}}{\rm{o}}{\rm{r}}\,{\rm{t}}{\rm{h}}{\rm{e}}\,{\rm{T}}\, \mbox{-} \,{\rm{m}}{\rm{a}}{\rm{z}}{\rm{e}}\\ 20 & {\rm{o}}{\rm{t}}{\rm{h}}{\rm{e}}{\rm{r}}{\rm{w}}{\rm{i}}{\rm{s}}{\rm{e}}\,.\end{array}$$. ${F}_{i}^{pc}$ and ${F}_{k}^{a}$ are sets containing respectively ${\bar{t}}_{i}$ and ${\bar{t}}_{k}$, the arrival times of all spikes fired by place cell *i* and action neuron *k*. Spiking behaviour is stochastic and follows an inhomogeneous Poisson process with parameter *λ*_*j*_(*u*_*j*_(*t*)), which depends on the membrane potential at time *t*. In particular,

3 $${\lambda }_{j}({u}_{j}(t))={\lambda }_{0}\,\exp (\frac{{u}_{j}(t)-\theta }{{\rm{\Delta }}u}),$$

where *λ*_0_ is the maximum firing rate, Δ*u* regulates randomness of the spiking behaviour and *θ* = 16 mV is the spiking threshold. For simplicity, the resting potential is set to 0. The biologically realistic value of the membrane potential can be retrieved through a translation and does not affect the dynamics of the network^33^.

**Table 3:** Parameter values.

	Radial arm	T-maze	Open Field
*w*_*min*_	1	1	1
*w*_*max*_	5	5	3
*w*_*in*_	2	3	2
*η*_ACh_	0.001	0.1	0.002
*η*_DA_	0.01	0.3	0.01

#### Discrete model

In the radial maze, there are only eight possible actions to take from the initial position. There are *N* = 8 neurons, each coding for a different arm. These neurons are connected through inhibitory synapses: *w*^*lat*^ = −250. This connectivity scheme ensures that, given enough time, one neuron will inhibit all others and be substantially more active. Other parameters were set to: *λ*_0_ = 100 Hz, Δ*u* = 0.5 mV.

#### Continuous model

Action neurons represent different directions in the Cartesian plane. Specifically, each action neuron *j* represents direction **a**_**j**_, where **a**_**j**_ = *a*_0_(sin(*θ*_*j*_), cos(*θ*_*j*_)), with ${\theta }_{j}=\frac{2j\pi }{N}$, *N* = 40 and *a*_0_ = 0.08. The lateral connectivity between action neuron *k* and action neuron *j* is defined as follows

4 $${w}_{jk}^{lat}=\frac{{w}_{-}}{N}+{w}_{+}\frac{f(\,j,k)}{Z},$$

where Z is a normalizing factor, *w*_–_ = −300, *w*_+_ = 100 and *f* is a lateral connectivity function, which is symmetric, positive and increases monotonically with the similarity of the actions. In particular, $f(j,k)=(1-{\delta }_{jk}){e}^{\psi \cos ({\theta }_{j}-{\theta }_{k})}$, with *ψ* = 20. Neurons therefore excite each other when they have a similar tuning, and depress otherwise. This ensure that only a few similarly tuned action neurons are active at any given time, making the trajectory of the agent smooth and consistent. Other parameters were set to: *λ*_0_ = 60 Hz, Δ*u* = 2 mV.

### Action selection

The action selection process determines the decision to take, based on the firing rates of the action neurons. The activity of action neuron *j* is approximated by filtering spike train *Y*_*j*_ with kernel *γ*:

5 $${\rho }_{j}(t)=({Y}_{j}\circ \gamma )(t),$$

where ${Y}_{j}={\sum }_{{\bar{t}}_{j}\in {F}_{j}^{a}}\,\delta (t-{\bar{t}}_{j})$ and $\gamma (t)=\frac{{e}^{\frac{-t}{{\tau }_{\gamma }}}-{e}^{\frac{-t}{{\nu }_{\gamma }}}}{{\tau }_{\gamma }-{\nu }_{\gamma }}{\rm{\Theta }}(t)$, with *τ*_*γ*_ = 50 ms and *ν*_*γ*_ = 20 ms.

#### Discrete model

Decisions in the discrete case are taken only at the end of the trial. When a time limit *T*_*max*_ = 5 s has been reached, the action neuron with maximum firing rate is selected. In the unlikely case two neurons exhibit exactly the same firing rate at the end of trial, the winning neuron is chosen at random. The agent then enters the arm associated with the winning neuron. All activity is reset before the onset of the next trial.

#### Continuous model

In the continuous case, actions are taken continuously, at every timestep *t*. The action selection process thus determines **a**(*t*), the action to take at time *t*. If each action neuron *j* represents direction **a**_*j*_ and has an estimated firing rate *ρ*_*j*_(*t*), then the action **a**(*t*) is the average of all the directions encoded, weighted by their respective firing rates

6 $${\bf{a}}(t)=\frac{1}{N}\,\sum _{j}\,{\rho }_{j}(t){{\bf{a}}}_{j},$$

where *N* = 40 is the total number of action neurons. This decision making mechanism allows the agent to move in any direction, making the action space effectively continuous. A large number of action neurons allows for higher the accuracy of the navigation and action selection.

### Navigation details

#### Continuous model

Once action **a**(*t*) has been determined, the update for the position of the agent is

7 $${\rm{\Delta }}{\bf{x}}(t)=\{\begin{array}{cc}{\bf{a}}(t), & {\rm{i}}{\rm{f}}\,{\bf{x}}({\rm{t}}+1)\,{\rm{w}}{\rm{i}}{\rm{t}}{\rm{h}}{\rm{i}}{\rm{n}}\,{\rm{t}}{\rm{h}}{\rm{e}}\,{\rm{b}}{\rm{o}}{\rm{u}}{\rm{n}}{\rm{d}}{\rm{a}}{\rm{r}}{\rm{i}}{\rm{e}}{\rm{s}}.\\ d\cdot {\bf{u}}({\bf{x}}(t)) & {\rm{o}}{\rm{t}}{\rm{h}}{\rm{e}}{\rm{r}}{\rm{w}}{\rm{i}}{\rm{s}}{\rm{e}}\end{array}$$

The agent therefore normally moves with instantaneous velocity **a**(*t*). When the agent tries to surpass the limits of the field, it is instantly bounced back by a distance *d* = 0.01. The unit vector **u**(**x**(t)) points in the direction opposite to the boundary. To avoid large boundary effects, the feed-forward weights between place cells on the boundaries and action neurons that code for a direction *a*_*j*_ outside of the field are set to zero.

The agent is free to explore the environment for a maximum duration of *T*_*max*_. If it finds the reward at a time *t*_*rew*_ < *T*_*max*_, the trial is terminated earlier, precisely at time *t* = *T*_*rew*_ + 300 ms. The extra time mimics consummatory behavior, navigation is thus paused during this interval (i.e. place cells activity is set to zero). The effect of the inter-trial interval is modelled by resetting all activity.

T-maze - continuous model: When used in the task, the reward is located in the right arm of the maze. Specifically, we consider the reward to be found whenever the agent crosses the vertical line *x*_*r*_ = 1 a.u. The maximum duration of a trial is *T*_*max*_ = 5 s, but the trial ends whenever the agent enters one of the arms (whenever the agent crosses either the vertical line *x*_*r*_ = 1 or the vertical line *x*_*l*_ = −1). When in the stem, the available actions are restricted only to upwards movements (angle between $\theta \in [\frac{\pi }{4},\frac{3\pi }{4}]$). When in the top part of the maze, only horizontal movements are allowed (angle between $\theta \in [-\frac{\pi }{4},\frac{\pi }{4}]\cup [-\frac{3\pi }{4},\frac{5\pi }{4}]$).

Open field - continuous model: For the first 20 trials, the reward can be found in the circular goal area centred in *c*_1_ = (1.5, 1.5) with radius *r*_1_ = 0.3. In trials 21 to 40, the goal area moves to centre *c*_2_ = (−1.5, −1.5), but maintains the same shape and size. If the open field has obstacles, the agent is not allowed to cross them and is therefore pushed back, similarly to what happens with the walls. In this case, the goal area is initially centred in *c*_1_ = (0, 1.5), and then moved to *c*_2_ = (0, −1.5). The maximum duration of a trial is *T*_*max*_ = 15 s. This maximum duration of a trial *T*_*max*_ was chosen so that the agent could discover the reward in the first few trials (Fig. 2A), its value is not intended to have behavioural or biological meaning.

### Sequentially neuromodulated plasticity (sn-Plast)

The synaptic weights between place cells and action neurons play a fundamental role in defining a policy for the agent. Plasticity is essential for the agent to learn to navigate the open field and is implemented in a way that follows the experimental results presented in Brzosko *et al*. 2015 and 2017. The synaptic changes combine the modified STDP rule (Fig. 3) and an eligibility trace that allows for delayed updates.

In particular, the total weight update is:

8 $${\rm{\Delta }}{w}_{ji}(t)=\eta A((\sum _{{\bar{t}}_{i}\in {F}_{i}^{pc}}\,\sum _{{\bar{t}}_{j}\in {F}_{j}^{a}}\,W({\bar{t}}_{j}-{\bar{t}}_{i}))\circ \psi )(t),$$

where *η* is the learning rate, *A* emulates the effect of the different neuromodulators, *W* is the STDP window and *ψ* is the eligibility trace. ${F}_{i}^{pc}$ and ${F}_{j}^{a}$ are sets containing respectively ${\bar{t}}_{i}$ and ${\bar{t}}_{j}$, the arrival times of all spikes fired by place cell *i* and action neuron *j*.

The basic STDP window is

9 $$W(x)={e}^{-\frac{|x|}{\tau }},$$

with *τ* = 10 ms. This function is always symmetric and positive, but the sign of the final weight change is determined by the neuromodulators at the synapse:

10 $$A=\{\begin{array}{cc}-1 & -{\rm{D}}{\rm{A}},+{\rm{A}}{\rm{C}}{\rm{h}}\\ 0 & -{\rm{D}}{\rm{A}},-{\rm{A}}{\rm{C}}{\rm{h}}\\ 1 & +{\rm{D}}{\rm{A}},\pm {\rm{A}}{\rm{C}}{\rm{h}}.\end{array}$$

Dopamine is assumed to be released simultaneously in all synapses whenever a reward is delivered. All weight changes are gated by neuromodulation (*A* = 0 when all neuromodulators are absent). The learning rate *η* also depends on neuromodulators (see Table 3 for specific values):

11 $$\eta =\{\begin{array}{cc}{\eta }_{{\rm{A}}{\rm{C}}{\rm{h}}} & -{\rm{D}}{\rm{A}},+{\rm{A}}{\rm{C}}{\rm{h}}\\ 0 & -{\rm{D}}{\rm{A}},-{\rm{A}}{\rm{C}}{\rm{h}}\\ {\eta }_{{\rm{D}}{\rm{A}}} & +{\rm{D}}{\rm{A}},\pm {\rm{A}}{\rm{C}}{\rm{h}}.\end{array}$$

The weight change due to STDP is convoluted with an eligibility trace *ψ*, modelled as an exponential decay

12 $$\psi (t)={e}^{-\alpha \frac{t}{{\tau }_{e}}}{\rm{\Theta }}(t),$$

with *τ*_*e*_ = 2 s and $$\alpha =\{\begin{array}{cc}1 & +{\rm{D}}{\rm{A}}\\ 0 & -{\rm{D}}{\rm{A}}\end{array}$$.

The eligibility trace keeps track of the active synapses and allows for a delayed update of the synaptic strength. The timescale of the eligibility trace *τ*_*e*_ determines the length of the rewarding path learned: a shorter timescale favours shorter paths. Variable *α* in the exponent acts as a flag and ensures that the eligibility trace is active with dopamine only (*α* = 1).

When no interaction with acetylcholine was assumed (−ACh), the weights were potentiated only at the end of the trial, in the case that the agent found the reward (*A* = 1, *α* = 1). They were left unchanged otherwise (*A* = 0). If acetylcholine was present throughout the task (+ACh), the weights were updated online (*A* = −1, *α* = 0). When no reward was found before the end of the trial, weights were depressed. Otherwise, they were potentiated retroactively (*A* = 1, *α* = 1).

### Dopamine-modulated standard asymmetric STDP curve

We also compared our symmetric learning windows to standard asymmetric STDP curves. The total weight update with this rule is

13 $${\rm{\Delta }}{w}_{ji}(t)=\eta B((\sum _{{\bar{t}}_{i}\in {F}_{i}^{pc}}\,\sum _{{\bar{t}}_{j}\in {F}_{j}^{a}}\,{W}_{2}({\bar{t}}_{j}-{\bar{t}}_{i}))\circ \psi )(t),$$

where *η* = 0.01 is the learning rate, *W*_2_ is the STDP window (equation 14) and *ψ* is the eligibility trace (equation 12). B gates all synaptic changes until the end of the trial: $$B=\{\begin{array}{cc}1 & {\rm{a}}{\rm{t}}\,{\rm{t}}{\rm{h}}{\rm{e}}\,{\rm{e}}{\rm{n}}{\rm{d}}\,{\rm{o}}{\rm{f}}\,{\rm{t}}{\rm{h}}{\rm{e}}\,{\rm{t}}{\rm{r}}{\rm{i}}{\rm{a}}{\rm{l}}\\ 0 & {\rm{d}}{\rm{u}}{\rm{r}}{\rm{i}}{\rm{n}}{\rm{g}}\,{\rm{e}}{\rm{x}}{\rm{p}}{\rm{l}}{\rm{o}}{\rm{r}}{\rm{a}}{\rm{t}}{\rm{i}}{\rm{o}}{\rm{n}}\end{array}$$. ${F}_{i}^{pc}$ and ${F}_{j}^{a}$ are sets containing ${\bar{t}}_{i}$ and ${\bar{t}}_{j}$ respectively, the arrival times of all spikes fired by place cell *i* and action neuron *j*. The spike timing plasticity rule was implemented as follows:

14 $${W}_{2}(s)=\{\begin{array}{cc}{A}_{pre-post}{e}^{-\frac{s}{\tau }} & {\rm{i}}{\rm{f}}\,s > 0\\ \frac{1}{2}({A}_{pre-post}+{A}_{post-pre}) & {\rm{i}}{\rm{f}}\,s=0\\ {A}_{post-pre}{e}^{\frac{s}{\tau }} & {\rm{i}}{\rm{f}}\,s < 0\end{array}$$

The integral of the learning window determines if the agent learns, unlearns or does not learn. We therefore considered four different parameter sets: (i) positive integral (*A*_*pre*–*post*_ = 1, *A*_*post*–*pre*_ = −0.5); (ii) negative integral (*A*_*pre*–*post*_ = 0.5, *A*_*post*–*pre*_ = −1); zero integral with either (iii) positive *A*_*pre*–*post*_ (standard STDP window; *A*_*pre*–*post*_ = 0.5, *A*_*post*–*pre*_ = −0.5) or (iv) negative *A*_*post*–*pre*_ (inverted STDP window; *A*_*pre*–*post*_ = −0.5, *A*_*post*–*pre*_ = 0.5). The time constant was identical for the two sides of the window and was taken to be *τ* = 10 ms. We ran 1000 simulations for each parameter set.

### Dynamic reward signal

We compared sn-Plast to a learning rule gated by a dynamic reward signal. This learning rule is similar to the one used in the control simulations (−ACh), but the weight change here is scaled by the dynamic reward signal $\rho (tr)=R(tr)-\bar{R}(tr)$. Here, *R*(*tr*) is the value of the reward received during trial *tr* and $\bar{R}(tr)$ is the moving average reward. In our simulations, we assumed that *R*(*tr*) = 1 if the agent reaches the rewarding area before the end of the trial, *R*(*tr*) = 0 otherwise. The moving average reward is calculated as $\bar{R}(tr)=\beta R(tr)+(1-\beta )\bar{R}(tr-1)$, with $\bar{R}(1)=R(1)$. In Fig. 6, we used *β* = 0.75. Here, *β* regulates the timescale of integration of the average reward. The higher *β*, the shorter the timescale. The weight update for simulations with dynamic reward signal is:

15 $${\rm{\Delta }}{w}_{ji}(t)=\eta B\rho (tr)((\sum _{{\bar{t}}_{i}\in {F}_{i}^{pc}}\,\sum _{{\bar{t}}_{j}\in {F}_{j}^{a}}\,W({\bar{t}}_{j}-{\bar{t}}_{i}))\circ \psi )(t),$$

where *η* = 0.01, *tr* is the current trial and B gates all synaptic changes until the end of the trial:$$B=\{\begin{array}{cc}1 & {\rm{a}}{\rm{t}}\,{\rm{t}}{\rm{h}}{\rm{e}}\,{\rm{e}}{\rm{n}}{\rm{d}}\,{\rm{o}}{\rm{f}}\,{\rm{t}}{\rm{h}}{\rm{e}}\,{\rm{t}}{\rm{r}}{\rm{i}}{\rm{a}}{\rm{l}}\\ 0 & {\rm{d}}{\rm{u}}{\rm{r}}{\rm{i}}{\rm{n}}{\rm{g}}\,{\rm{e}}{\rm{x}}{\rm{p}}{\rm{l}}{\rm{o}}{\rm{r}}{\rm{a}}{\rm{t}}{\rm{i}}{\rm{o}}{\rm{n}}\end{array}$$. The eligibility trace *ψ* (equation 12) is active only when the dynamic reward signal is delivered at the end of the trial:

16 $$\alpha =\{\begin{array}{cc}1 & {\rm{a}}{\rm{t}}\,{\rm{t}}{\rm{h}}{\rm{e}}\,{\rm{e}}{\rm{n}}{\rm{d}}\,{\rm{o}}{\rm{f}}\,{\rm{t}}{\rm{h}}{\rm{e}}\,{\rm{t}}{\rm{r}}{\rm{i}}{\rm{a}}{\rm{l}}\\ 0 & {\rm{d}}{\rm{u}}{\rm{r}}{\rm{i}}{\rm{n}}{\rm{g}}\,{\rm{e}}{\rm{x}}{\rm{p}}{\rm{l}}{\rm{o}}{\rm{r}}{\rm{a}}{\rm{t}}{\rm{i}}{\rm{o}}{\rm{n}}.\end{array}$$

### Negative feedback signal

We also compared our neuromodulated learning rule to a dopamine-modulated rule with negative feedback. In this set of simulations, we assumed that whenever the agent reaches the location of an omitted reward it receives a negative feedback that inverts the sign of the learning window induced by dopamine. The weight update for simulations with negative feedback is:

17 $${\rm{\Delta }}{w}_{ji}(t)=\eta {A}_{feedback}((\sum _{{\bar{t}}_{i}\in {F}_{i}^{pc}}\,\sum _{{\bar{t}}_{j}\in {F}_{j}^{a}}\,W({\bar{t}}_{j}-{\bar{t}}_{i}))\circ \psi )(t),$$

where *η* = 0.01, *A*_*feedback*_ = 1 when the new reward is found, *A*_*feedback*_ = −1 if the agent navigates to the old reward location and *A*_*feedback*_ = 0 otherwise. The eligibility trace *ψ* (equation 12) is active only when the feedback signal is delivered at the end of the trial (equation 16).

## Electronic supplementary material

Supplementary material

## References

[1] Schultz W, Dayan P, Montague PR (1997). A neural substrate of prediction and reward. Science.

[2] Schultz W (1997). Dopamine neurons and their role in reward mechanisms. Current Opinion in Neurobiology.

[3] Montague, P. R., Dayan, P. & Sejnowski, T. J. A framework for mesencephalic dopamine systems based on predictive Hebbian learning. *Journal of Neuroscience* **16**, 1936–1947, 10.1.1.156.635 (1996). 10.1523/JNEUROSCI.16-05-01936.1996 PMC6578666 8774460

[4] Inglis FM, Day JC, Fibiger HC (1994). Enhanced acetylcholine release in hippocampus and cortex during the anticipation and consumption of a palatable meal. Neuroscience.

[5] Inglis FM, Fibiger HC (1995). Increases in hippocampal and frontal cortical acetylcholine release associated with presentation of sensory stimuli. Neuroscience.

[6] Giovannini MG (2001). Effects of novelty and habituation on acetylcholine, GABA, and glutamate release from the frontal cortex and hippocampus of freely moving rats. Neuroscience.

[7] Ceccarelli I (1999). Effects of novelty and pain on behavior and hippocampal extracellular ACh levels in male and female rats. Brain Research.

[8] Thiel CM, Huston JP, Schwarting RKW (1998). Hippocampal acetylcholine and habituation learning. Neuroscience.

[9] Acquas E, Wilson C, Fibiger HC (1996). Conditioned and unconditioned stimuli increase frontal cortical and hippocampal acetylcholine release: effects of novelty, habituation, and fear. Journal of Neuroscience.

[10] Stancampiano R, Cocco S, Cugusi C, Sarais L, Fadda F (1999). Serotonin and acetylcholine release response in the rat hippocampus during a spatial memory task. Neuroscience.

[11] Fadda F, Cocco S, Stancampiano R (2000). Hippocampal acetylcholine release correlates with spatial learning performance in freely moving rats. Neuroreport.

[12] Ragozzino ME, Unick KE, Goldt PE, Mcgaugh JL (1996). Hippocampal acetylcholine release during memory testing in rats: Augmentation by glucose. Psychology.

[13] Ragozzino ME, Pal SN, Unick K, Stefani MR, Gold PE (1998). Modulation of hippocampal acetylcholine release and spontaneous alternation scores by intrahippocampal glucose injections. Journal of neuroscience.

[14] Kametani H, Kawamura H (1990). Alterations in acetylcholine release in the rat hippocampus during sleep-wakefulness detected by intracerebral dialysis. Life Sciences.

[15] Dayan P (2012). Twenty-five lessons from computational neuromodulation. Neuron.

[16] Markram H, Lübke J, Frotscher M, Sakmann B (1997). Regulation of synaptic efficacy by coincidence of postsynaptic aps and epsps. Science.

[17] Bi G-Q, Poo M-M (1998). Synaptic modifications in cultured hippocampal neurons: dependence on spike timing, synaptic strength, and postsynaptic cell type. Journal of neuroscience.

[18] Zhang J-C, Lau P-M, Bi G-Q (2009). Gain in sensitivity and loss in temporal contrast of STDP by dopaminergic modulation at hippocampal synapses. Proceedings of the National Academy of Sciences of the United States of America.

[19] Edelmann E, Leßmann V, Brigadski T (2014). Pre-and postsynaptic twists in bdnf secretion and action in synaptic plasticity. Neuropharmacology.

[20] Yang K, Dani JA (2014). Dopamine D1 and D5 receptors modulate spike timing-dependent plasticity at medial perforant path to dentate granule cell synapses. Journal of Neuroscience.

[21] Brzosko Z, Schultz W, Paulsen O (2015). Retroactive modulation of spike timing-dependent plasticity by dopamine. eLife.

[22] Brzosko Z, Zannone S, Schultz W, Clopath C, Paulsen O (2017). Sequential neuromodulation of hebbian plasticity offers mechanism for effective reward-based navigation. eLife.

[23] Izhikevich EM (2007). Solving the distal reward problem through linkage of STDP and dopamine signaling. Cerebral Cortex.

[24] Legenstein R, Pecevski D, Maass W (2008). A learning theory for reward-modulated spike-timing-dependent plasticity with application to biofeedback. PLoS Computational Biology.

[25] Pan W-X (2005). Dopamine cells respond to predicted events during classical conditioning: evidence for eligibility traces in the reward-learning network. Journal of Neuroscience.

[26] Suri RE, Schultz W (1999). A neural network model with dopamine-like reinforcement signal that learns a spacial delayed response task. Neuroscience.

[27] Florian RV (2007). Reinforcement learning through modulation of spike-timing-dependent synaptic plasticity. Neural Computation.

[28] Teles-Grilo Ruivo, L. M. & Mellor, J. R. Cholinergic modulation of hippocampal network function. *Frontiers in Synaptic Neuroscience* **5**, 10.3389/fnsyn.2013.00002 (2013). 10.3389/fnsyn.2013.00002 PMC3726829 23908628

[29] Foster, D. J., Morris, R. G. & Dayan, P. A model of hippocampally dependent navigation, using the temporal difference learning rule. *Hippocampus* **10**, 1–16, 10.1002/(SICI)1098-1063 (2000). 10.1002/(SICI)1098-1063(2000)10:1<1::AID-HIPO1>3.0.CO;2-1 10706212

[30] Vasilaki E, Frémaux N, Urbanczik R, Senn W, Gerstner W (2009). Spike-based reinforcement learning in continuous state and action space: when policy gradient methods fail. PLoS Comput Biol.

[31] Frémaux N, Sprekeler H, Gerstner W (2010). Functional requirements for reward-modulated spike-timing-dependent plasticity. Journal of Neuroscience.

[32] Fremaux N, Sprekeler H, Gerstner W (2013). Reinforcement learning using a continuous time actor-critic framework with spiking neurons. PLoS Computational Biology.

[33] Gerstner, W. & Kistler, W. M. *Spiking Neuron Models*: *Single Neurons*, *Populations*, *Plasticity*, 1 edn. (Cambridge University Press, 2002).

[34] Marrosu F (1995). Microdialysis measurement of cortical and hippocampal acetylcholine release during sleep-wake cycle in freely moving cats. Brain Research.

[35] Kempter R, Gerstner W, Van Hemmen JL (1999). Hebbian learning and spiking neurons. Physical Review E.

[36] Rescorla, R. A. & Wagner, A. W. A theory of Pavlovian conditioning: Variations in the effectiveness of reinforcement and nonreinforcement. In Black, A. H. & Prokasy, W. F. (eds) *Classical Conditioning II*: *Current Research and Theory*, chap. 3, 64–99 (Appleton-Century-Crofts, New York, 1972).

[37] Pavlov IP (1927). Conditioned reflexes: An investigation of the physiological activity of the cerebral cortex.

[38] Thorndike, E. L. Animal intelligence: Experimental studies (Macmillan, 1911).

[39] Sutton RS, Barto AG (1998). Reinforcement Learning: An Introduction.

[40] Lisman J (1989). A mechanism for the hebb and the anti-hebb processes underlying learning and memory. Proceedings of the National Academy of Sciences.

[41] McNamara CG, Tejero-Cantero Á, Trouche S, Campo-Urriza N, Dupret D (2014). Dopaminergic neurons promote hippocampal reactivation and spatial memory persistence. Nature Neuroscience.

[42] Lisman JE, Grace AA (2005). The hippocampal-VTA loop: Controlling the entry of information into long-term memory. Neuron.

[43] Li S, Cullen WK, Anwyl R, Rowan MJ (2003). Dopamine-dependent facilitation of LTP induction in hippocampal CA1 by exposure to spatial novelty. Nature Neuroscience.

[44] Otmakhova, N., Duzel, E., Deutch, A. Y. & Lisman, J. The Hippocampal-VTA Loop: The Role of Novelty and Motivation in Controlling the Entry of Information into Long-Term Memory. In Baldassarre, G. & Mirolli, M. (eds) *Intrinsically Motivated Learning in Natural and Artificial Systems*, 235–254, 10.1007/978-3-642-32375-1_10 (Springer Berlin Heidelberg, Berlin, Heidelberg, 2013).

[45] Tran AH (2008). Dopamine D1 Receptor Modulates Hippocampal Representation Plasticity to Spatial Novelty. Journal of Neuroscience.

[46] Ihalainen JA, Riekkinen P, Feenstra MGP (1999). Comparison of dopamine and noradrenaline release in mouse prefrontal cortex, striatum and hippocampus using microdialysis. Neuroscience Letters.

[47] Atherton LA, Dupret D, Mellor JR (2015). Memory trace replay: The shaping of memory consolidation by neuromodulation. Trends in Neurosciences.

[48] De Lavilléon G, Lacroix MM, Rondi-Reig L, Benchenane K (2015). Explicit memory creation during sleep demonstrates a causal role of place cells in navigation. Nature neuroscience.

[49] Hasselmo ME (2006). The role of acetylcholine in learning and memory. Current opinion in neurobiology.

[50] Deiana S, Platt B, Riedel G (2011). The cholinergic system and spatial learning. Behavioural Brain Research.

[51] Easton A, Douchamps V, Eacott M, Lever C (2012). A specific role for septohippocampal acetylcholine in memory?. Neuropsychologia.

[52] Boddeke EWGM, Enz A, Shapiro G (1992). SDZ ENS 163, a selective muscarinic M1 receptor agonist, facilitates the induction of long-term potentiation in rat hippocampal slices. European Journal of Pharmacology.

[53] Huerta PT, Lisman JE (1995). Bidirectional synaptic plasticity induced by a single burst during cholinergic theta oscillation in CA1 *in vitro*. Neuron.

[54] Ovsepian SV, Anwyl R, Rowan MJ (2004). Endogenous acetylcholine lowers the threshold for long-term potentiation induction in the CA1 area through muscarinic receptor activation: *In vivo* study. European Journal of Neuroscience.

[55] Shinoe T, Matsui M, Taketo MM, Manabe T (2005). Modulation of synaptic plasticity by physiological activation of M1 muscarinic acetylcholine receptors in the mouse hippocampus. Journal of Neuroscience.

[56] Buchanan KA, Petrovic MM, Chamberlain SE, Marrion NV, Mellor JR (2010). Facilitation of long-term potentiation by muscarinic M1 receptors is mediated by inhibition of SK channels. Neuron.

[57] Connor S, Maity S, Roy B, Ali DW, Nguyen PV (2012). Conversion of short-term potentiation to long-term potentiation in mouse CA1 by coactivation of -adrenergic and muscarinic receptors. Learning & Memory.

[58] Digby GJ (2012). Novel allosteric agonists of M1 muscarinic acetylcholine receptors induce brain region-specific responses that correspond with behavioral effects in animal models. Journal of Neuroscience.

[59] Dennis SH (2016). Activation of muscarinic M1 acetylcholine receptors induces long-term potentiation in the hippocampus. Cerebral Cortex.

[60] Adams SV, Winterer J, Müller W (2004). Muscarinic signaling is required for spike-pairing induction of long-term potentiation at rat Schaffer collateral-CA1 synapses. Hippocampus.

[61] Sugisaki E, Fukushima Y, Tsukada M, Aihara T (2011). Cholinergic modulation on spike timing-dependent plasticity in hippocampal CA1 network. Neuroscience.

[62] Sugisaki E, Fukushima Y, Fujii S, Yamazaki Y, Aihara T (2016). The effect of coactivation of muscarinic and nicotinic acetylcholine receptors on LTD in the hippocampal CA1 network. Brain Research.

[63] Scheiderer CL (2006). Sympathetic sprouting drives hippocampal cholinergic reinnervation that prevents loss of a muscarinic receptor-dependent long-term depression at CA3-CA1 synapses. Journal of Neuroscience.

[64] Volk LJ, Pfeiffer BE, Gibson JR, Huber KM (2007). Multiple Gq-coupled receptors converge on a common protein synthesis-dependent long-term depression that is affected in fragile X syndrome mental retardation. Journal of Neuroscience.

[65] Dickinson BA (2009). A novel mechanism of hippocampal ltd involving muscarinic receptor-triggered interactions between ampars, grip and liprin-*α*. Molecular Brain.

[66] Jo J (2010). Muscarinic receptors induce LTD of NMDAR EPSCs via a mechanism involving hippocalcin, AP2 and PSD-95. Nature Neuroscience.

[67] Kamsler A, McHugh TJ, Gerber D, Huang SY, Tonegawa S (2010). Presynaptic M1 muscarinic receptors are necessary for mGluR long-term depression in the hippocampus. Proceedings of the National Academy of Sciences of the United States of America.

[68] Picciotto MR, Higley MJ, Mineur YS (2012). Acetylcholine as a neuromodulator: cholinergic signaling shapes nervous system function and behavior. Neuron.

[69] Pepeu G, Giovannini MG (2004). Changes in acetylcholine extracellular levels during cognitive processes. Learning & memory.

[70] Flicker C, Geyer MA (1982). Behavior during hippocampal microinfusions. II. Muscarinic locomotor activation. Brain Research Reviews.

[71] Tzavos A, Jih J, Ragozzino ME (2004). Differential effects of M1 muscarinic receptor blockade and nicotinic receptor blockade in the dorsomedial striatum on response reversal learning. Behavioural Brain Research.

[72] McCool MF, Patel S, Talati R, Ragozzino ME (2008). Differential involvement of M1-type and M4-type muscarinic cholinergic receptors in the dorsomedial striatum in task switching. Neurobiology of Learning and Memory.

[73] Ragozzino ME, Jih J, Tzavos A (2002). Involvement of the dorsomedial striatum in behavioral flexibility: Role of muscarinic cholinergic receptors. Brain Research.

[74] Dong, Z. *et al*. Hippocampal long-term depression mediates spatial reversal learning in the Morris water maze. *Neuropharmacology* **64**, 65–73, 10.1016/j.neuropharm.2012.06.027 (2013). 10.1016/j.neuropharm.2012.06.027 22732443

[75] Doya K (2002). Metalearning and neuromodulation. Neural Networks.

[76] Yu AJ, Dayan P (2005). Uncertainty, neuromodulation, and attention. Neuron.

[77] Hasselmo ME (1999). Neuromodulation: Acetylcholine and memory consolidation. Trends in Cognitive Sciences.

