# Supplementary material for: Acetylcholine-modulated plasticity in reward-driven navigation: a computational study
Source: Sci Rep. 2018 Jun 21;8:9486. doi: 10.1038/s41598-018-27393-2 (PMC6013476; doi:10.1038/s41598-018-27393-2)
Supplement: Supplementary file 1 — Supplementary material [file 41598_2018_27393_MOESM1_ESM.pdf]

Supplementary material for "Acetylcholine-modulated plasticity in reward-driven navigation: a computational study."

Sara Zannone, [1] Zuzanna Brzosko,[2], Ole Paulsen,[2] and Claudia Clopath[1,*]

[1] Imperial College London, Department of Bioengineering, South Kensington Campus, London, United Kingdom
[2] University of Cambridge, Department of Physiology, Development and Neuroscience, Physiological Laboratory, Cambridge, United Kingdom
[*] c.clopath@imperial.ac.uk

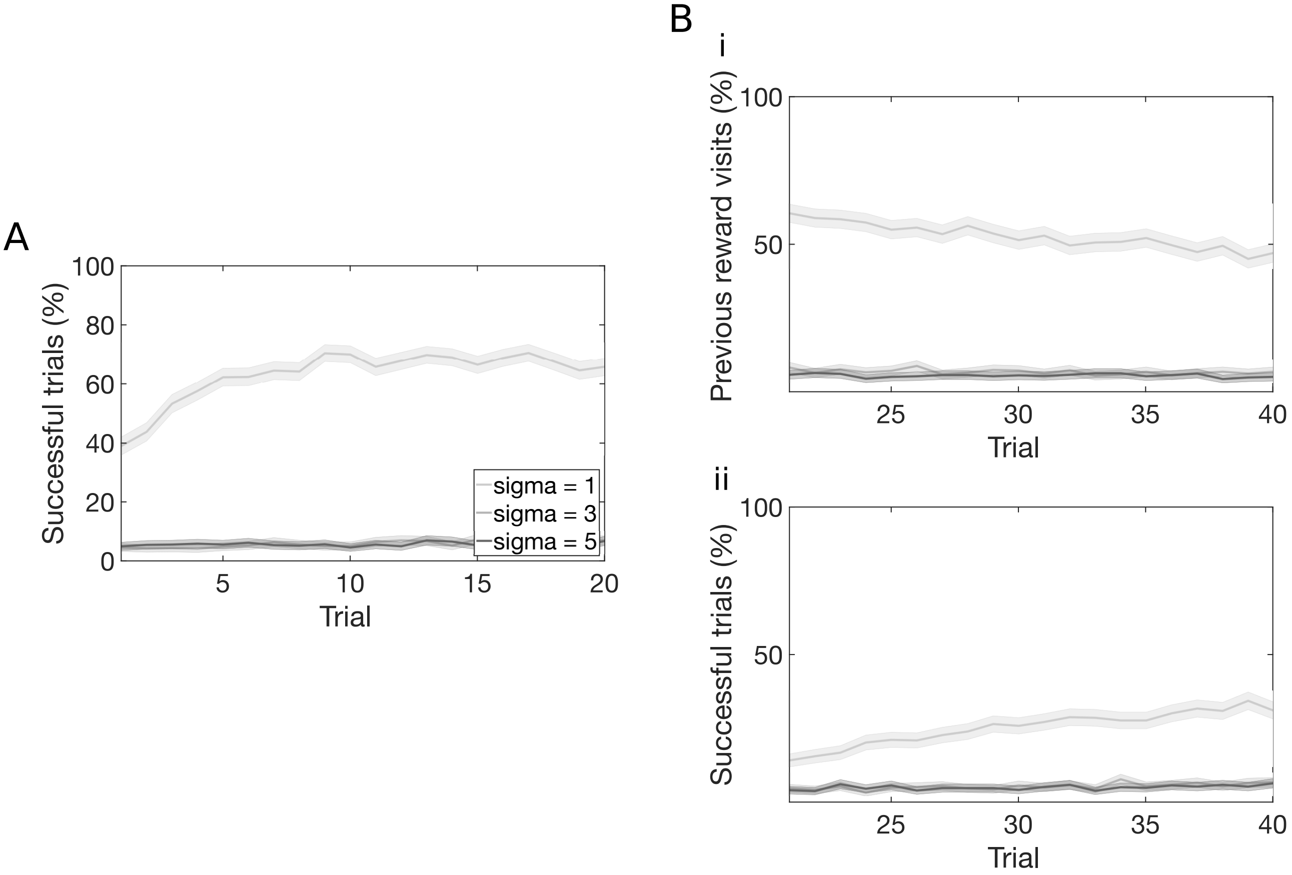

Supplementary figure 1. Noise in the neural activity. Gaussian noise is added to the neural activity of -ACh agents. Simulations are run under three different conditions: with added Gaussian noise of zero mean and standard deviations $\sigma = 1$ (light grey), $\sigma = 3$ (grey) and $\sigma = 5$ (dark grey). The task is the same as in Fig 6 and Fig 7. A. Percentage of successful simulations across trials 1-20. Agents start each trial from the centre of the open field and have to navigate to the top-right corner. Even with small noise, the performance is compromised. B. Trials 21-40. The reward is moved to the opposite side of the open field. i) Percentage of agents visiting the old reward location. ii) Percentage of successful simulations as a function of the trial number. The shaded area represents the 95% confidence interval of the sample mean. A little noise helps the performance in the second half of the trial, but it does not really help unlearning and is substantially worse than +ACh agents (Fig 6 and Fig 7).

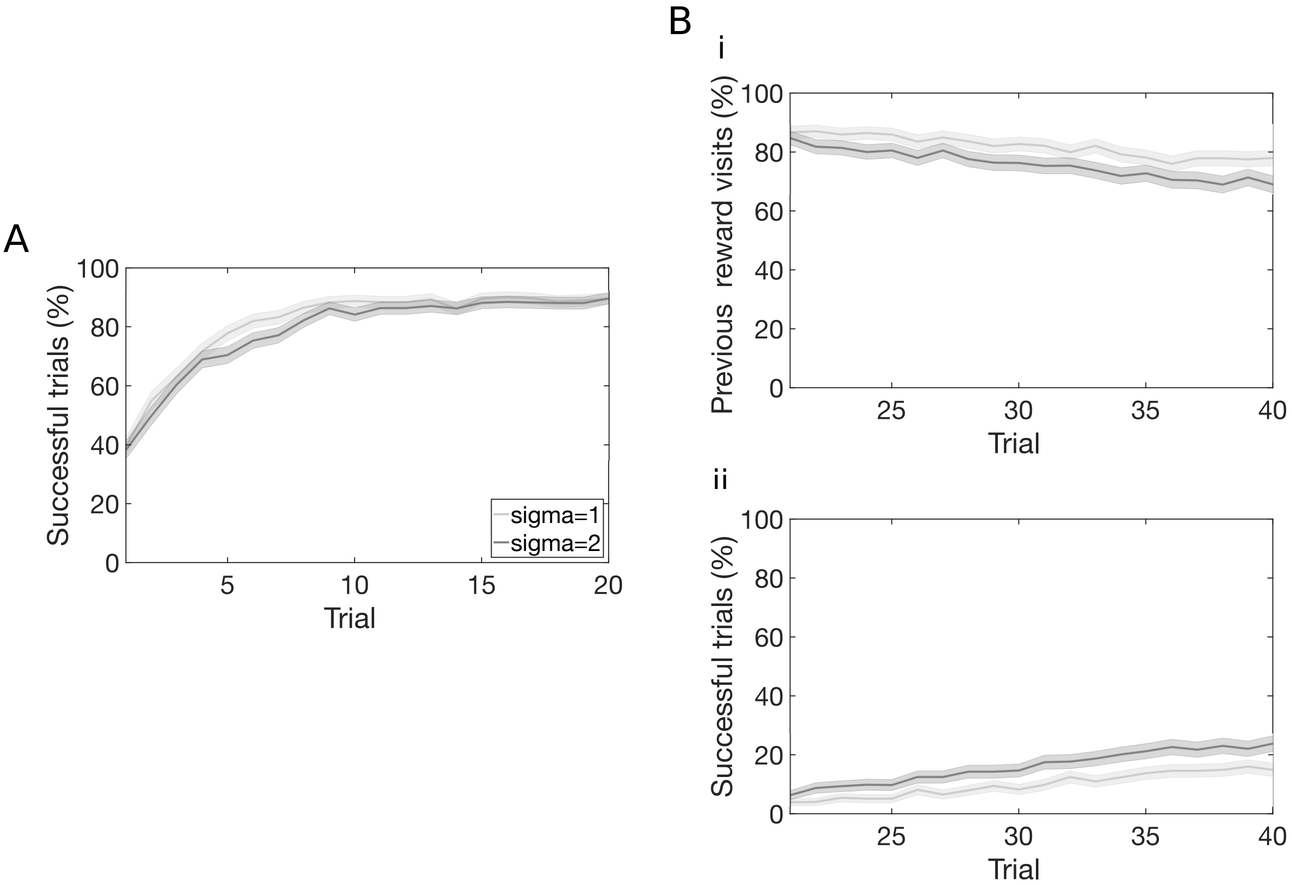

Supplementary figure 2. Noise in the synaptic weights. Gaussian noise is added to the synaptic weights of -ACh agents. Simulations are run under two conditions: with added Gaussian noise of zero mean and standard deviations $\sigma = 1$ (light grey) and $\sigma = 2$ (dark grey). A. Percentage of successful simulations across trials 1-20. Agents start each trial from the centre of the open field and have to navigate to the top-right corner, the noise does not disrupt learning much. B. Trials 21-40. The reward is moved to the opposite side of the open field. i) Percentage of agents visiting the old reward location. ii) Percentage of successful simulations as a function of the trial number. The shaded area represents the 95% confidence interval of the sample mean. The noise helps the performance very slightly in the second half of the trial, but it is still inferior to sn-Plast (Fig 6 and Fig 7).

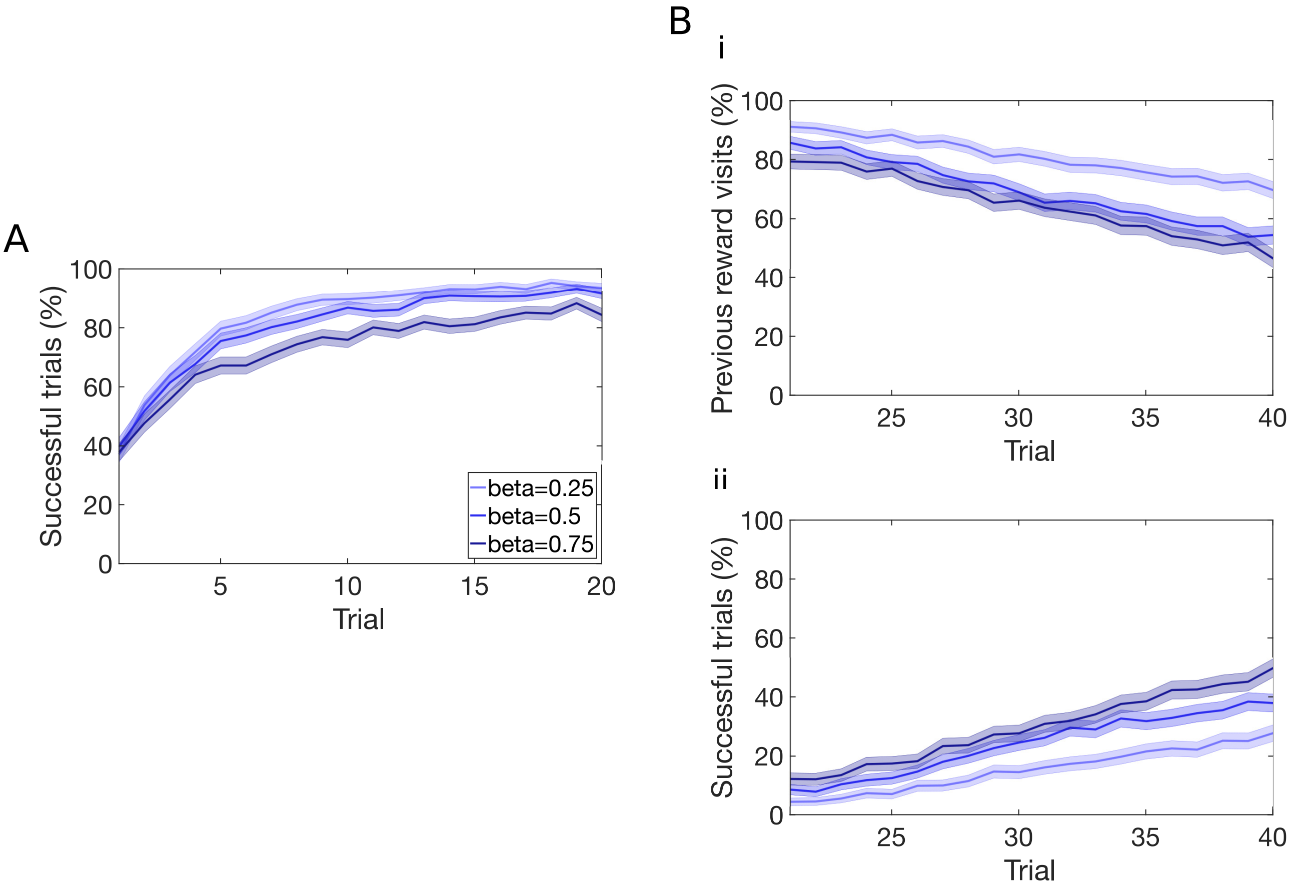

Supplementary figure 3. Dynamic reward signal with different timescales of integration for the average reward. Simulations are run under three conditions: short timescale (dark blue, $\beta = 0.75$), medium timescale (blue, $\beta = 0.5$) and long timescale (light blue, $\beta = 0.25$). A. Percentage of successful simulations across trials 1-20. Agents start each trial from the centre of the open field and have to navigate to the top-right corner (task as in Figure 6A). Longer timescales are better at learning, because they lead to increased synaptic potentiation. B. Trials 21-40. The reward is moved to the opposite side of the open field (task as in Figure 6B). i) Percentage of agents visiting the old reward location. ii) Percentage of successful simulations as a function of the trial number. The shaded area represents the 95% confidence interval of the sample mean. Here, shorter timescales are actually advantageous, as they pick up the change in the environment more promptly and lead to increased depression. Although agents show some behavioural flexibility, their performance is not nearly as good as +ACh agents (Fig. 6).
